# Artificial Intelligence-Driven SELEX Design of Aptamer Panels for Urinary Multi-Biomarker Detection in Prostate Cancer: A Systematic and Bibliometric Review

**DOI:** 10.3390/biomedicines13122877

**Published:** 2025-11-25

**Authors:** Ayoub Slalmi, Nabila Rabbah, Ilham Battas, Ikram Debbarh, Hicham Medromi, Abdelmjid Abourriche

**Affiliations:** 1 Laboratory of Biology and Health (LBS), Faculty of Sciences Ben M’Sik, Hassan II University of Casablanca, Casablanca 20670, Morocco; ayoub.slalmi-etu@etu.univh2c.ma; 2 Research Foundation for Development and Innovation in Science and Engineering (FRDISI), Casablanca 20250, Morocco; 3 Graduate School of Biomedical Engineering and Health Techniques (SUPTECH-SANTÉ), Mohammedia 28830, Morocco; 4 Laboratory of Complex Cyber Physical Systems (LCCPS), ENSAM, Hassan II University of Casablanca, Casablanca 20000, Morocco

**Keywords:** prostate cancer, urinary extracellular vesicles, Aptamers, SELEX, artificial intelligence, microRNA, multiplex diagnostics, systematic review, risk of bias, bibliometric analysis

## Abstract

**Background/Objectives:** The limited specificity of prostate-specific antigen (PSA) drives unnecessary biopsies in prostate cancer (PCa). Urinary extracellular vesicles (uEVs) provide a non-invasive reservoir of tumor-derived nucleic acids and proteins. Aptamers selected by SELEX enable highly specific capture, and artificial intelligence (AI) can accelerate their optimization. This systematic review evaluated AI-assisted SELEX for urine-derived and exosome-enriched aptamer panels in PCa detection. **Methods:** Systematic searches of PubMed, Scopus, and Web of Science (1 January 2010–24 August 2025; no language restrictions) followed PRISMA 2020 and PRISMA-S. The protocol is registered on OSF (osf.io/b2y7u). After deduplication, 1348 records were screened; 129 studies met the eligibility criteria, including 34 (26.4%) integrating AI within SELEX or downstream refinement. Inclusion required at least one quantitative metric (dissociation constant K_d_, SELEX cycles, limit of detection [LoD], sensitivity, specificity, or AUC). Risk of bias was appraised with QUADAS-2 (diagnostic accuracy studies) and PROBAST (prediction/machine learning models). **Results:** AI-assisted SELEX workflows reduced laboratory enrichment cycles from conventional 12–15 to 5–7 (≈40–55% relative reduction) and reported K_d_ values spanning low picomolar to upper nanomolar ranges; heterogeneity and inconsistent comparators precluded pooled estimates. Multiplex urinary panels (e.g., PCA3, TMPRSS2:ERG, miR-21, miR-375, EN2) yielded single-study AUCs between 0.70 and 0.92 with sensitivities up to 95% and specificities up to 88%; incomplete 2 × 2 contingency reporting prevented bivariate meta-analysis. LoD reporting was sparse and non-standardized despite several ultralow claims (attomolar to low femtomolar) on nanomaterial-enhanced platforms. Pre-analytical variability and absent threshold prespecification contributed to high or unclear risk (QUADAS-2). PROBAST frequently indicated high risk in participants and analysis domains. Across the included studies, lower K_d_ and reduced LoD improved analytical detectability; however, clinical specificity and AUC were predominantly shaped by pre-analytical control (matrix; post-DRE vs. spontaneous urine) and prespecified thresholds, so engineering gains did not consistently translate into higher diagnostic accuracy. **Conclusions:** AI-assisted SELEX is a promising strategy for accelerating high-affinity aptamer discovery and assembling multiplex urinary panels for PCa, but current evidence is early phase, heterogeneous, and largely single-center. Priorities include standardized uEV processing, complete 2 × 2 diagnostic reporting, multicenter external validation, calibration and decision impact analyses, and harmonized LoD and K_d_ reporting frameworks.

## 1. Introduction

Current prostate cancer diagnostic pathways still hinge on serum PSA prompting targeted or systematic biopsy, although PSA’s limited specificity and frequent benign or inflammatory elevations drive avoidable procedures and overdiagnosis [1]. This persistent performance ceiling has accelerated interest in non-invasive matrices capable of refining pre-biopsy risk stratification. Urine, readily obtainable, repeatable, and directly influenced by prostatic secretions, retains molecular and extracellular vesicle (EV) cargo often diluted or confounded in blood [1,2]. EV fractions protect proteins, lipids, and nucleic acids that mirror tumor phenotypes and preserve their integrity during handling, creating a resilient substrate for multiplex biosensing [3,4]. Methodological advances (size exclusion, microfluidic immunocapture, nano-flow cytometry, label-free electrical transduction) reinforce analytical yield and scalability [5]. Early demonstrations include urinary detection of Engrailed-2 using hybrid aptamer-antibody HCR-ELONA and graphene transistor platforms, and PCA3 via aptamer-based electrochemical or impedimetric sensors, confirming feasibility in native, minimally processed urine [2,6,7]. SELEX-derived aptamers combine high affinity and specificity with batch-consistent chemical synthesis and modular functionalization suited to miniaturized multiplex devices, even within complex urinary matrices [3,8]. Collectively, the convergence of PSA’s diagnostic ceiling, the biochemical richness of urine (including its EV compartment), and the engineering flexibility of aptamer systems delineates a translational opportunity for multiplex urinary signatures to refine biopsy triage and reduce unnecessary invasive sampling [1,2,3,4,5,6,7,8].

Beyond biomarker-oriented evidence, several foundational studies on SELEX optimization, aptamer engineering, and biosensing technologies have shaped the methodological landscape exploited in this review [3,4,5,6,7,8,9,10,11,12,13,14,15,16,17,18,19,20,21,22,23,24,25,26,27,28,29,30,31].

To date, no systematic review has quantitatively synthesized the impact of AI integration in SELEX workflows for urinary aptamer-based prostate cancer diagnostics.

Despite recognition of PSA’s constraints, clinical practice remains slow to operationalize robust adjunctive tools, and current pre-biopsy decision algorithms still underperform in discriminating indolent from clinically significant disease [9,10,11,12,13,14]. A key unmet need is an integrated panel that couples analytically stable urinary EV targets with rapid, low-cost signal transduction to deliver actionable post-PSA risk refinement. Existing single-analyte or dual-marker assays seldom exploit the full composite information space (protein conformation states, small RNA cargo, and low-abundance transcription factors) [7,8,15,16]. Aptamer platforms can bridge this gap: iterative SELEX optimization (including negative selection against benign prostatic hyperplasia matrices), tunable dissociation constants into the low-nanomolar or sub-nanomolar range, and orthogonal labeling strategies (electroactive tags, enzymatic amplification, nanomaterial conjugation) enable layered specificity while maintaining manufacturability [1,2,3,5,6]. Strategic development priorities now include (i) standardization of pre-analytical urine handling for EV integrity, (ii) head-to-head benchmarking of aptamer-EV signatures versus established kallikrein or exosomal RNA panels, (iii) analytical-clinical bridging studies reporting harmonized metrics (AUC with confidence intervals, paired sensitivity/specificity, decision-curve analysis, and calibration), and (iv) prospective validation within MRI-integrated pathways [10,11,12,13,14]. Framing these steps within transparent reporting (STARD, QUADAS-2/PROBAST) will accelerate regulatory credibility and facilitate clinical adoption.

Primary objective (Population/Index/Comparator/Outcomes/Study designs):

Population: individuals undergoing evaluation for suspected prostate cancer prior to biopsy.
Index tests: urinary aptamer-based assays (single or multiplex), including those incorporating extracellular vesicle (EV) cargo.
Comparator: histopathological biopsy (reference standard) and, where available, established adjunct tests (e.g., kallikrein panels, exosomal RNA assays).
Outcomes: diagnostic accuracy metrics (sensitivity, specificity, AUC with 95% confidence intervals, likelihood ratios, diagnostic odds ratio, HSROC parameters).
Study designs: diagnostic accuracy investigations (prospective, retrospective, proof-of-concept with extractable 2 × 2 data).

Secondary objectives: (i) quantify SELEX performance metrics (dissociation constant K_d_, number of enrichment cycles, time-to-hit, stability/chemistry) and relate them to assay accuracy; (ii) assess the influence of pre-analytical factors (urine handling, EV isolation) and analytical choices (aptamer chemistry/modifications, transduction modality, AI-driven optimization) on diagnostic yield; (iii) benchmark multiplex aptamer-EV signatures versus established serum and urine panels using harmonized endpoints (AUC with 95% CIs, paired sensitivity/specificity, LR+, LR−, DOR, calibration, decision-curve analysis); (iv) appraise risk of bias and applicability (diagnostic studies: QUADAS-2; prediction models, if present: PROBAST) and report completeness using a focused SELEX-AI checklist.

Tertiary objective: characterize bibliometric trends (publication trajectory, collaboration networks, thematic clustering spanning EV analytics, electrical transduction, and AI-guided SELEX).

Methodological contribution: A PRISMA-compliant systematic review integrated with a quantitative bibliometric landscape; searches (PRISMA-S) will span multiple databases and gray literature sources; records will be de-duplicated, screened in duplicate with agreement statistics (Cohen’s κ), and extracted via a pre-specified template covering diagnostic, SELEX, and implementation endpoints (turnaround time, cost per test, internal vs. external validation); meta-analysis will apply Reitsma’s bivariate model with HSROC plotting, plus sensitivity and heterogeneity analyses and publication bias assessment (Deeks’ test where applicable); bibliometrics (VOSviewer/Bibliometrix) will map keyword co-occurrence, temporal overlays, venues, and collaborations to identify thematic clusters (EV analytics, electrical transduction, AI-guided SELEX) and field dynamics.

This review will be conducted and reported in accordance with PRISMA 2020. PRISMA-S statement: All search strategies and information sources will be fully documented and reported in accordance with the PRISMA-S extension.

## 2. Methodology

### 2.1. Registration, Sources, and Search Strategy (PRISMA/PRISMA-S)

The protocol was prospectively registered (OSF: osf.io/b2y7u, accessed on 3 November 2025), before any study selection; no deviations were introduced. The Supplementary Materials Checklist is provided to ensure full transparency of search, screening, and extraction procedures. The covered period spans 1 January 2010 to 24 August 2025 (inclusive). The data lock was set at 2025-08-24T23:47Z (UTC) and this timestamp is reported consistently (Methods, PRISMA flow legend, Appendix). The following three bibliographic databases were queried: PubMed/MEDLINE, Scopus, and Web of Science Core Collection. Trial registries, preprints (medRxiv, bioRxiv), and patent databases were excluded by strategy (structural heterogeneity, peer-review robustness objective).

The multiblock search combined controlled vocabulary (MeSH, Emtree, WoS categories) and free-text terms covering aptamers/SELEX, urinary biomarkers (including extracellular vesicles/exosomes), prostate cancer, and diagnostic terminology (sensitivity, specificity, ROC, AUC, K_d_, LoD). Truncations, orthographic variants, phrase searching, and proximity operators (NEAR/3) were used to maximize recall while limiting thematic drift. Minor harmonizations (exosom*, specific*, aptamer*) preceded execution. The strategy underwent internal peer review by an independent information specialist; no substantive modification was required (syntax-only adjustments). Execution timeline (UTC): PubMed 2025-08-24T21:05Z; Scopus 2025-08-24T21:32Z; Web of Science 2025-08-24T22:10Z.

Across the three predefined query blocks (Q1–Q3), we retrieved a total of 2951 records: Q1 (core intersection) 2467, Q2 (biomarker/diagnostic extension) +83, and Q3 (performance-focused: sensitivity, specificity, AUC, ROC) 401. Following hierarchical deduplication (normalized DOI, then composite key: title/author/year, then fuzzy title similarity with a threshold ≥0.90 and manual adjudication; retention priority: PubMed > Scopus > Web of Science), 1348 unique records advanced to title and abstract screening. All exportable metadata (title, abstract, DOI, authors, affiliations, author keywords, controlled terms, year, source, document type) were consolidated into a unified workspace, with each procedural element (query log, field mapping, merge rules) fully version-tracked to ensure reproducibility.

**Figure 1:**
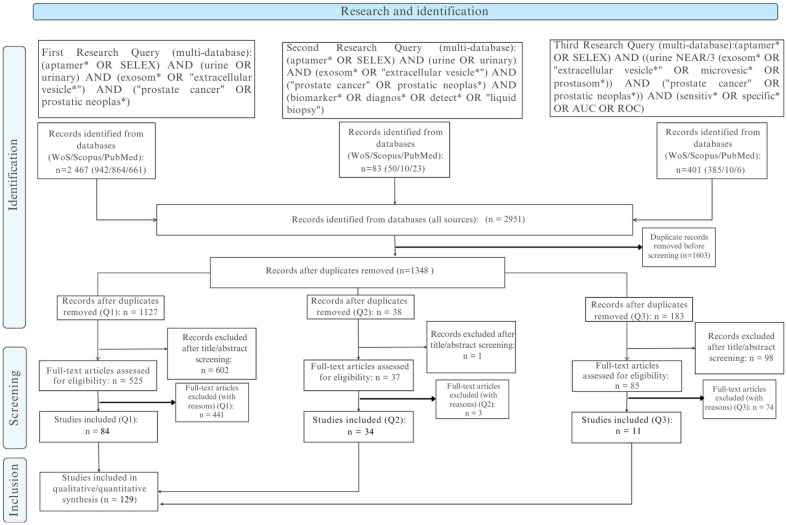
PRISMA 2020 flow diagram of the study selection process (PubMed, Scopus, Web of Science).

The report follows PRISMA 2020 (doi:10.1136/bmj.n71) and PRISMA-S (doi:10.5195/jmla.2021.962). Figure 1 (PRISMA flow) includes the note: “Six included full texts lacked extractable quantitative metrics and were retained qualitatively; they were excluded from pooled analyses.”

### 2.2. Eligibility Criteria and Selection

The bibliometric metadata are presented in Table 1, after initial screening (1348 records), 701 title/abstract exclusions left 647 full texts. Of these 647, 518 were excluded for categorized reasons (Table 2), yielding 135 full-text studies included. Of the 135, 129 had extractable quantitative data (diagnostic, analytical, and/or affinity) and constitute the “evaluable” subset; six (4 from Q1, 2 from Q2) were retained qualitatively without metric integration. Post-deduplication distribution by block: Q1 1127; Q2 38; Q3 183. Full texts included Q1 88 (84 evaluable), Q2 36 (34 evaluable), Q3 11 (all evaluable).

Inclusion criteria: original studies using urinary samples (cell-free whole urine, urinary extracellular vesicles/exosomes, exfoliated cells) and/or aptamers derived from a SELEX process applied to diagnosis or risk stratification of prostate cancer, reporting at least one quantitative measure (diagnostic performance, analytical metric, affinity constant). Exclusions: secondary literature, purely therapeutic applications without diagnostic metric, platform descriptions lacking a relevant cohort/specimen, non-urinary or non-prostate matrices, absence of an aptamer (non-conforming index test), non-extractable data (absence of Se, Sp, AUC, LoD, K_d,_ or equivalents), redundant cohorts without added value, retraction/inaccessibility.

Screening was performed in duplicate (piloted form); disagreements resolved by consensus. Inter-reviewer reliability: observed agreement 93.2%; kappa (κ) = 0.689 (95% CI 0.628–0.746); prevalence index 0.75; bias index 0.05. No retrospective author contact (pre-specified).

### 2.3. PICOS Framework

Population: Men evaluated for prostate cancer (initial biopsy, active surveillance, confirmed cases), native or derivative urinary samples (EV, exfoliated cells); for selection/affinity studies: cell lines or cellular structures contextually relevant to the prostate urinary axis (where diagnostic intent is explicit).

Intervention/Index test: Aptamer-based tests, sensors, or signatures (electrochemistry EIS/DPV/FET, fluorescence/amplification, SPR, microfluidic platforms, nanopore, nanomaterial hybrids) and SELEX processes (cell-SELEX, protein-SELEX, hybrids, in silico refinements).

Comparators: Histopathology (biopsy) and/or standard-of-care biomarkers (PSA, %free PSA, PHI, PCA3, TMPRSS2:ERG, mpMRI) or clinically established thresholds; for purely analytical performance: internal standards or spiked samples (not treated as diagnostic comparators).

Outcomes: Diagnostic performance (Se, Sp, AUC, LR+, LR−, DOR, PPV, NPV when 2 × 2 data available), analytical metrics (LoD, LoQ, dynamic range, assay time, stability, reproducibility), affinity (K_d_, log_10_(K_d_), ΔG), SELEX parameters (cycles, counter-selection).

Designs: Experimental studies, analytical validations, diagnostic accuracy studies (prospective, retrospective, cross-sectional, case–control), engineering, or optimization of aptamers with urinary prostate anchoring.

Exclusions: Reviews/secondary reports, purely therapeutic approaches, in silico without empirical validation, non-urinary matrices, absence of quantifiable metric.

### 2.4. Data Extraction and Target Variables

Two reviewers independently extracted (structured template) the following: publication metadata; study type; matrix (native urine, artificial urine, buffer, urinary EV, exfoliated cells); cohort description (total size, cases, controls/benign); pre-analytical context if reported (collection, digital rectal exam status, storage); index platform (electrochemical subtype, optical, SPR, amplification, microfluidic, hybrid).

Diagnostic: Se, Sp, AUC, PPV, NPV (only if 2 × 2 data), LR+, LR−, DOR (2 × 2 required), 95% CI, cut-off definition (pre-specified vs. derived), validation level (internal simple, cross-validation, external cohort), reproducibility (intra/inter-assay), stability (storage, matrix perturbations).

Analytical: LoD, LoQ (if explicit), dynamic range (min–max, converted to molar where possible), calibration coefficient (R^2^), span (RangeMax/RangeMin), ratio (RangeMin/LoD) to estimate effective sensitivity exploitation, assay time (sample-to-result), and total workflow duration.

Affinity/SELEX: Target (class: protein, cell surface, nucleic, metabolite, EV), aptamer identifier, length (nt), chemical modifications (2′F, LNA, PEG, thiol, biotin, locked bases), selection mode (cell, protein, hybrid, in silico), cycles, counter-selection strategy, K_d_ (units verified), log_10_(K_d_ [M]), $\Delta G=R\cdot T\cdot ln\left(K_{d}\right)$ with default T = 298 K; recalculated at experimental T (277 or 310 K) when explicitly reported. Approximate values (digitized) marked “*” and excluded from high-confidence sensitivity analyses. Qualitative non-quantifiable affinities categorized (E1–E5) to map gaps.

No imputation; unavailable field is “NR”. AUCs arising solely from internal cross-validation are annotated to distinguish from external validations.

### 2.5. Risk of Bias Assessment

Diagnostic accuracy studies were assessed with QUADAS-2 (patient selection, index test, reference standard, flow/timing) plus applicability concerns. Multivariate signatures or predictive modeling approaches (if present) were examined with PROBAST (participants, predictors, outcome, analysis). Non-randomized methodological comparisons with potential confounding were contextually described using ROBINS-I (qualitative only, not replacing QUADAS-2 judgments). An internal SELEX transparency framework (cycles, enrichment kinetics, counter-selection, library complexity management, algorithmic/AI augmentation, validation modality) generated reporting flags (adequate vs. partial/insufficient).

Dual assessment with calibration phase (n = 10). No composite score; “uncertain” only when textual insufficiency is explicit. High-risk domains or applicability concerns feed sensitivity exclusions. GRADE-DTA not applied (high extrinsic heterogeneity).

### 2.6. Evidence Synthesis Strategy and Bibliometric Analyses

Bibliometrics: A distinct corpus (n = 756) was constituted (after pre-analytical exclusion of 136 out-of-scope or incomplete records) for mapping: annual output (2010–2025*), source dispersion, co-author networks (fractional counting), international collaborations, keyword co-occurrence. Tools: VOSviewer (v1.x) and Bibliometrix (R 2024). Citation counts normalized to “citations per year” to mitigate recency bias. The compound annual growth rate (CAGR) is calculated as

CAGR=V2025V2010115−1.

Diagnostic synthesis: Metrics (Se, Sp, AUC, LR+, LR−, DOR) are reported descriptively. Quantitative pooling is considered only if ≥5 studies evaluate the same biomarker-platform construct in a clinically comparable context (population spectrum, matrix, threshold). Modeling: bivariate (Reitsma) with hierarchical summary ROC (HSROC), 95% CI, and prediction regions. Continuity correction (0.5) for zero cells. No formal publication bias assessment if <10 studies per cell. Formulas:

$${LR}^{+}=\frac{Se}{1-Sp},{LR}^{-}=\frac{1-Se}{Sp},DOR=\frac{{LR}^{+}}{{LR}^{-}}.$$

DOR excludes studies lacking 2 × 2 data. AUCs derived exclusively from internal self-validation are not aggregated (optimism bias mitigation).

Affinity: Distribution of K_d_ (high-confidence subset) by target class: median, IQR, range; ΔG reported at 298 K and at assay temperature when provided (expected effect: more negative ΔG at higher T when $ln\left(K_{d}\right)<0$. No meta-aggregation (non-harmonizable variability: ionic strength, buffer, format).

Analytical: LoD harmonized to molar when conversions are unambiguous; dynamic range presented (lower bound, upper bound), span, and ratio (RangeMin/LoD) to estimate effective sensitivity leverage. Narrative synthesis is due to format and unit diversity.

Missing data handling: “NR” without imputation. Pre-specified sensitivity analyses: exclusion (i) multi-domain high risk of bias studies; (ii) assays on artificial matrices without validation on native urine.

Limitations: Exclusion of gray literature (risk of publication bias), under-reporting of pre-analytics (collection, storage conditions), matrix and platform heterogeneity limiting quantitative aggregations, absence of kinetic parameters (k_on, k_off) impairing fine mechanistic interpretation of ΔG.

Eligibility criteria encompassed original investigations reporting urinary biomarkers (cell-free urine, urinary extracellular vesicles, or exfoliated urinary cells) and/or aptamer- or SELEX-based approaches applied to prostate cancer diagnosis or risk stratification. Studies were required to provide experimental data or quantitative diagnostic performance measures (e.g., sensitivity, specificity, AUC, or affinity constants such as K_d_). Exclusion criteria comprised secondary or overview literature (reviews, editorials, commentaries), therapeutic aptamer applications without a diagnostic component, methodological or platform papers lacking a clinical or biological cohort, and studies with non-extractable or incomplete outcome data.

Two reviewers independently screened records in a dual-review workflow at the title/abstract and full-text stages using a piloted, standardized form. Discrepancies were resolved through consensus. Agreement between reviewers was quantified using Cohen’s κ. At the title/abstract stage (n = 1348 unique records after de-duplication), the overall inclusion rates were 9.6% for Reviewer 1 and 15.0% for Reviewer 2. Observed agreement was 93.2%, with Cohen’s κ = 0.689 (95% CI 0.628–0.746), indicating substantial inter-reviewer reliability. The prevalence index (0.75) reflected the expected predominance of exclusions, while the bias index (0.05) indicated minimal systematic imbalance between reviewers.

From 2951 initial records (Scopus, Web of Science, and PubMed combined), 1603 duplicates were removed, leaving 1348 records for screening. Of these, 701 were excluded at the title/abstract stage and 647 underwent full-text assessment. A total of 518 full texts were excluded (secondary literature, non-prostate focus, methodological platforms without diagnostic data, or insufficient extractable outcomes), yielding 129 studies included in the final synthesis. Detailed distributions of exclusion reasons are provided in Supplementary Table S1.

### 2.7. Software

**Figure 2:**
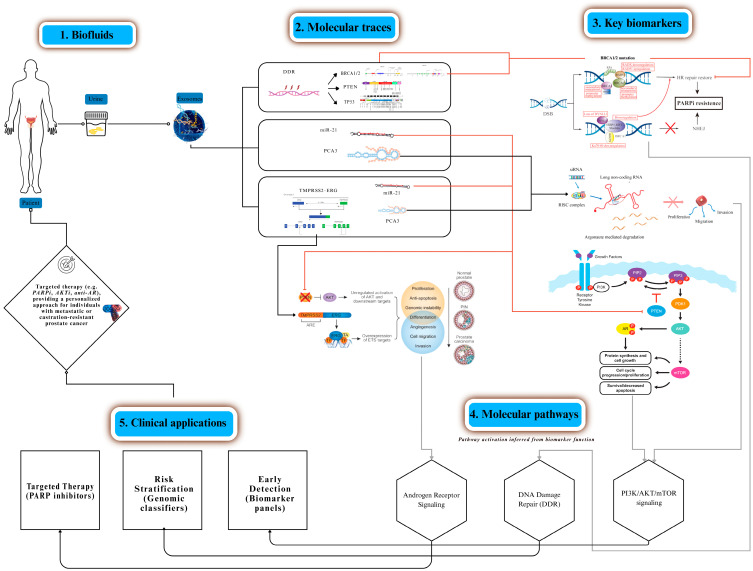
Schematic representation of urinary and exosomal biomarkers in prostate cancer. NOTE Figure 2: Urine (post-DRE) provides multiple fractions (exosomes, exfoliated cells, soluble phase) carrying molecular traces such as PCA3 [2], TMPRSS2:ERG [32,33], and exosomal miR-21 [8,34,35] that map to androgen receptor signaling and metastatic progression. Proteins such as PSA [9] and EN2 [1] add complementary diagnostic value, while exosome-based aptamer capture (e.g., CD63, CD9) improves biomarker recovery [36,37,38]. These analytes converge on key molecular pathways and clinically actionable processes supported by aptamer-based diagnostics and liquid-biopsy platforms [7,36,37,38,39]. Clinical applications include biomarker-guided early detection (PCA3, TMPRSS2:ERG), risk stratification via exosomal microRNA signatures (miR-21, miR-375) [8,34,35,40], and targeted therapies such as PARP inhibitors, ARSi, PI3K inhibitors, and PSMA-directed theranostics [15,16].

**Figure 3:**
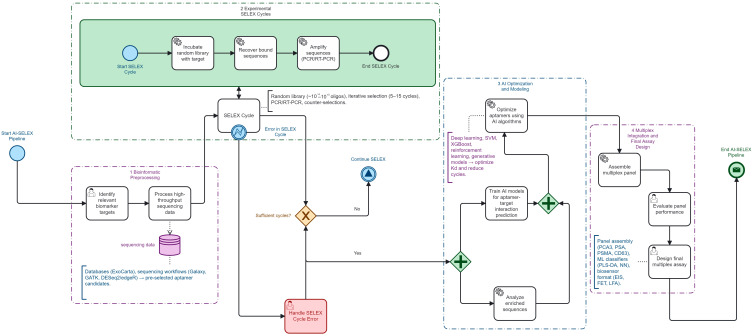
AI-SELEX pipeline from preprocessing to aptamer optimization and panel design.

All bibliometric analyses were performed using VOSviewer v1.6.20 (Leiden University, The Netherlands), Bibliometrix/Biblioshiny v4.2.2 (University of Naples, Italy), RStudio v2023.09 (Boston, MA, USA), and Microsoft Excel 365 (Microsoft Corporation, USA). Figure 2 was created using Canva Pro (Canva Pty Ltd., Sydney, Australia), and Figure 3 was created using Camunda Modeler v5.18 (Camunda Services GmbH, Berlin, Germany).

## 3. Urinary and Exosomal Biomarkers

### 3.1. Priority Targets (PCA3, TMPRSS2:ERG, EN2, PSA; ±miR-21/miR-375)

Urine has emerged as the preferred non-invasive matrix for prostate cancer because it directly captures prostatic secretions enriched in molecular cargo. Among the most validated targets are the lncRNA PCA3 [2], the TMPRSS2:ERG fusion transcript [32,33], the homeobox protein EN2 [1], and variants of PSA [9], complemented by exosomal miR-21 and miR-375 [34,35], Each marker contributes orthogonal information: PCA3 and TMPRSS2:ERG refine pre-biopsy stratification [2,32], EN2 enables direct urinary protein detection [1,36], PSA retains regulatory and clinical approval value [9], and microRNAs capture aggressive phenotypes, particularly bone-tropic disease [7,34,35]. Their joint integration maximizes sensitivity and specificity compared with single-analyte assays.

### 3.2. Biological Rationale for Urinary Multiplexing 

Urine fractions exosomes, exfoliated cells, and soluble analytes carry complementary biomarker classes, enabling multiplex panels that mitigate PSA’s specificity ceiling. Exosomes protect nucleic acids and proteins from degradation, mirror tumor phenotypes, and extend detection windows [7,15]. Orthogonality across fractions reduces false positives: fusion transcripts and lncRNAs are enriched in exfoliated cells [37,38], PSA and metabolites in soluble phase [38,39], and oncomiRs in vesicular cargo [8,34,35,40]. This biological compartmentalization supports multi-analyte biosensing strategies and justifies the design of aptamer panels capturing structurally diverse ligands under the same diagnostic pipeline.

### 3.3. Reported Performance of Aptamer Urinary Assays

Proof-of-concept assays highlight the feasibility of aptamer-based urinary diagnostics. Solution-gated graphene transistors achieved highly sensitive EN2 detection in urine [1]; electrochemical impedimetric and voltametric biosensors reliably quantified PCA3 transcripts [2,6]; and nanomaterial-based aptasensors improved PSA monitoring with sub-picogram limits of detection [9]. Recent advances also highlight the relevance of CD9-specific aptamer/MXene field-effect transistor platforms for highly sensitive detection of small extracellular vesicles, providing an additional benchmark for urinary EV-based assays [31]. More recent approaches extend to aptamer exosome hybrids that enable tumor theranostics by combining capture, signal amplification, and targeted delivery [15,16]. While these exemplars vary in platform and maturity, collectively they demonstrate that aptamers can achieve robust analytical performance and, when multiplexed, approach clinical translation thresholds.

## 4. AI and Bioinformatics Pipeline Steering SELEX

### 4.1. Pre-Processing and Target Nomination 

Upstream integration of omics and exosomal repositories is now central to target nomination in AI-guided SELEX. Transcriptomic resources help identify highly expressed oncogenic drivers [28], while curated extracellular vesicle databases highlight vesicular miRNAs and proteins with diagnostic relevance [23]. Proteomic mapping further delineates secreted biomarkers for panel inclusion [41], while foundational SELEX and post-SELEX studies on prostate cancer–specific aptamers and metastatic probes continue to inform target nomination workflows and molecular prioritization [42,43,44,45], additional post-SELEX refinements and cancer stem cell–oriented aptamer engineering provide methodological baselines that support the selection of high-specificity candidates [46,47]. Combined analyses that ensure multiplex aptamer panels capture orthogonal pathways rather than redundant signals [25,48,49,50,51].

### 4.2. AI for Design/Optimization 

Computational steering increasingly relies on machine-learning classifiers and structure–activity models that map sequence–structure–affinity relationships [29,52,53,54], together with deep-learning frameworks originally developed for genome-wide nucleic-acid binding prediction that can be transferred to aptamer design [55,56]. Diffusion-based generative architectures further explore novel candidate sequences beyond the experimental search space [57]. Reviews of computational nanomedicine pipelines and non-SELEX design strategies consistently report accelerated optimization of aptamer panels when AI is integrated into the workflow [22,53,55,58,59]. Hybrid pipelines that combine physics-informed or MD-derived descriptors with neural architectures achieve measurable gains in predicted binding affinity and specificity [55,56,57]. Multiparametric aptamer-based profiling frameworks further illustrate how advanced analytical pipelines can integrate multi-feature signatures for improved target discrimination and downstream prioritization [60,61,62,63,64]. RaptGen exemplifies the use of hybrid RNA–DNA aptamer design validated experimentally against viral proteins [65], while structure–activity models support rational prioritization of candidates in prostate and other cancers [52,54]. Overall, recent surveys emphasize that deep-learning foundational models applied at genome scale provide a scalable backbone for integrating high-throughput computation directly into SELEX and post-SELEX analyses [53,55,56].

### 4.3. Practical Integration (TensorFlow/PyTorch/Sklearn; Validation)

The overall workflow is summarized in Figure 3. Implementation in laboratory workflows is facilitated by open-source ecosystems such as TensorFlow for neural network training [17], PyTorch for modular deep learning applications [20], and scikit-learn for reproducible statistical pipelines [23]. Coupling these frameworks with experimental feedback accelerates enrichment efficiency [41], and pilot studies confirm that hybrid wet-lab/AI loops can yield multiplex aptamer panels suited for clinical translation.

Pre-processing integrates transcriptomic [28], exosomal [23], and proteomic [41] resources to identify non-redundant biomarkers [25]. Design and optimization apply ML [29], DL [30], RL [20], ensemble models [66], and genetic algorithms [22], with diffusion [55], nanomedicine reviews [56], hybrid predictors [67], APIPred [54], RaptGen [65], high-throughput reviews [53,55], and foundation models [56] extending discovery. Practical integration relies on TensorFlow [17], PyTorch [20], and scikit-learn [23], validated by hybrid AI-wet-lab pipelines [41].

## 5. Comparative Endpoints and Definitions

### 5.1. Diagnostic Performance Metrics

Diagnostic outcomes were standardized as area under the ROC curve (AUC), sensitivity (Se), specificity (Sp), and 95% confidence intervals (95% CI) where available. From these, we calculated positive and negative likelihood ratios (LR+, LR−) and the diagnostic odds ratio (DOR) to enable cross-platform comparability. These indices follow QUADAS-2 guidance and support synthesis via hierarchical summary ROC (HSROC) or bivariate meta-analysis when at least three comparable datasets were available.

### 5.2. SELEX-Derived Affinity and Selection Parameters

Aptamer engineering outputs were harmonized by recording the equilibrium dissociation constant (K_d_, nM), normalized to log_10_(K_d_ [M]), and, when possible, converted to Gibbs free energy ($\Delta G=R\cdot T\cdot ln(K_{d}).$ at 298 K). Additional descriptors included the number of enrichment cycles, total selection time, and the presence of counter-selection steps. Stability (thermal, storage, or reproducibility) and translatability (validation in patient-derived matrices beyond buffer or synthetic systems) were systematically captured to contextualize affinity metrics.

### 5.3. Operational Feasibility Indicators

Clinical translation was assessed through turnaround time (TAT), cost per test (directly reported or inferred from reagents/platform complexity), and external validation in independent cohorts. These parameters situate analytical performance within implementation constraints and map onto established technology readiness frameworks.

## 6. Bibliometric Landscape

### 6.1. Temporal Trends and Source Venues

The consolidated analytical corpus comprised 756 publications (2010–2025), derived from 892 initial records after 136 exclusions. Annual output rose modestly from 28 documents in 2010 to 34 in 2025, corresponding to a compound annual growth rate (CAGR) of 1.30%. At data lock (24 August 2025), the corpus had accumulated 31,610 citations (mean 41.83; median 21) and 52,762 cited references (mean 69.78; median 50). A total of 2956 disambiguated authors produced 5066 authorship instances (mean 6.70 per document; median 6), with 191 international collaborations (25.3%). The distribution by type was dominated by research articles (488; 64.6%) and reviews (234; 31.0%), with other formats being marginal. Citation indices confirmed a robustly cited core (h = 82; g = 142). Publications were dispersed across 314 source venues, with multidisciplinary nanotechnology and biosensor journals contributing the majority of highly cited works.

### 6.2. Keyword Co-Occurrence and Emerging Themes

Keyword co-occurrence mapping (VOSviewer, association strength normalization, full counting) was performed on author keywords with a minimum co-occurrence threshold of 2 (sensitivity map at threshold 1). The network contained twenty-four items, six clusters, and sixty-nine links (total link strength = 95). The central hub aptamer connected to six thematic modules:

(i) SELEX methodology (SELEX, cell-SELEX, G-quadruplex);
(ii) electrochemical biosensing platforms (electrochemical sensor, electrochemical impedance spectroscopy, differential/pulse voltammetry, screen-printed electrodes);
(iii) prostate cancer biomarkers (prostate cancer, PCA3, sarcosine, castration-resistant prostate cancer);
(iv) extracellular vesicles/exosome capture;
(v) imaging and physico-chemical tools (fluorescence imaging, atomic force microscopy);
(vi) chemical functionalization (chemical modification and related processes).

Notably, clinically relevant metrics such as LoD, K_d_, and AUC did not appear among the top author keywords, reflecting their underreporting in the primary literature. This reinforces the need to triangulate bibliometric mapping with systematic extraction of analytical performance data. Temporal overlays indicated a surge of terms related to AI-SELEX and multiplex biosensing after 2020, while earlier years clustered more strongly around classical SELEX and protein targets.

### 6.3. Country/Team Collaborations and Key Contributors

Co-authorship mapping highlighted a fragmented yet intensifying global network. European–East Asian consortia acted as central hubs bridging SELEX methodology with urinary biomarker applications. International collaborations accounted for one quarter of the corpus, but the overall density remained below adjacent diagnostic fields, suggesting opportunities for broader multicenter initiatives. Author leadership rotated over time from early SELEX pioneers to recent AI-SELEX contributors. Collectively, the bibliometric signal reflects a transition from isolated methodological reports toward networked, translational agendas.

**Figure 4:**
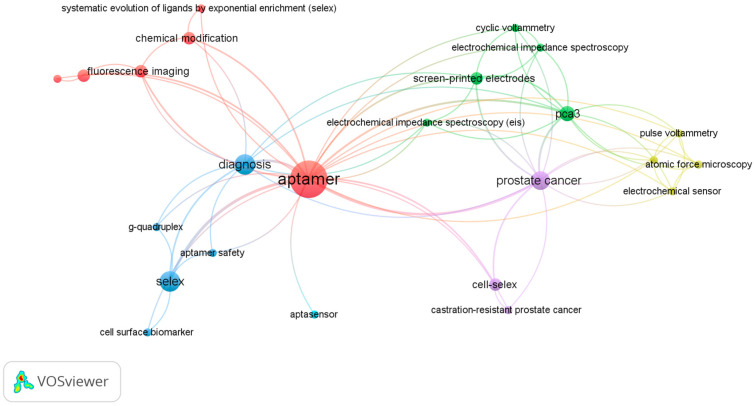
Bibliometric landscape of urinary aptamer research.

The bibliometric keyword structure is illustrated in Figure 4, which visualizes the major thematic clusters anchoring urinary aptamer research.

VOSviewer keyword co-occurrence map (author keywords, full counting, association strength normalization; threshold ≥ 2). The node aptamer anchors six thematic clusters as follows: (i) SELEX methodology, (ii) electrochemical biosensing platforms, (iii) prostate cancer biomarkers, (iv) extracellular vesicles, (v) imaging/physico-chemical tools, and (vi) chemical functionalization. Node size reflects keyword frequency; link thickness indicates co-occurrence strength; colors denote clusters detected by modularity optimization.

## 7. Results

### 7.1. Study Selection and Screening Agreement

The search (PubMed, Scopus, Web of Science) identified 2951 records. After removing 1603 duplicates, 1348 titles/abstracts were screened; 701 were excluded, leaving 647 full texts assessed. Of these, 518 were excluded for categorized reasons (Table 2; individual details DOI + code in Table 3), resulting in 135 full-text studies included. Of these 135, 129 had extractable quantitative data (diagnostic, analytical, and/or affinity) and constitute the “evaluable” subset; 6 were qualitative only (retained for narrative synthesis, excluded from quantitative analyses). Post-selection distribution by conceptual blocks is as follows: Q1 88 (84 evaluable), Q2 36 (34 evaluable), Q3 11 (all evaluable). Integration of an AI component in the SELEX workflow or downstream optimization was present in 34/129 studies (26.4%). Inter-reviewer agreement is the following: observed concordance 93.2%, κ = 0.689 (95% CI 0.628–0.746), consistent with substantial reliability [29,40,56]. Figure 1 (PRISMA) notes that six included texts without quantitative metrics were not incorporated into numerical syntheses.

### 7.2. Study Characteristics

Designs span early analytical validations (electrochemical platforms EIS/DPV/FET, ELONA/HCR, SPR), cell or protein binding studies (cell-SELEX, protein-SELEX, exosome/enrichment hybrids), and multiparametric diagnostic prototypes (combinations PCA3, TMPRSS2:ERG, EN2, miR-21, miR-375, sarcosine) [1,2,3,4,5,6,7,8,9,10,11,19,21,22,23,24,27,28,29,39,68,69,70,71,72,73,74]. Matrices include native urine (pre- or post-DRE), artificial urine (AUM/Surine™), urinary exosome fractions (uEV), and exfoliated cells. Maturity varies: many studies remain confined to artificial matrices or very small cohorts (often n < 30), limiting generalizability.

Table 4 presents the annual publication trends and citation distribution of the included studies, providing the bibliometric frame required before interpreting the analytical performance detailed in Table 5.

### 7.3. Analytical Performance (LoD, Dynamic Ranges)

Aptasensors exhibit limits of detection spanning from low nanomolar (enzyme-free assays) down to attomolar (graphene platforms for EN2) [1,2,3,5,75,76]. Structured examples (Table 5): EN2 on SGFET transistor: LoD 2.74 × 10^−18^ M (buffer) [1]; PCA3 (EIS AuNP-Aptamer): LoD 1.0 × 10^−15^ M (buffer), shifting to 2.0 × 10^−14^ M in AUM while retaining a dynamic range of 10^2−^10^4^ (RangeMin/LoD ratio) [2]; sarcosine apta-MIP (EIS): LoD 1.66 × 10–13 M across two distinct analytical segments [75]; HCR-ELONA EN2: LoD 3.40 × 10^−10^ M (buffer) and 2.69 × 10^−9^ M (AUM) with R^2^ ≥ 0.97 [5]. The extreme dispersion (often >10^9^ between LoD and upper bound) motivates an “effective dynamic utilization” metric (RangeMin/LoD; computed in Table 5).

### 7.4. Aptamer Affinity and SELEX Parameters

Most K_d_ values cluster from the high picomolar to the hundreds of nanomolar range (Table 6) [77,78], with log_10_(K_d_ [M]) and ΔG at 298 K computed via ΔG = R·T·ln(K_d_) [79,80]. Thermal adjustments (277 K and 310 K) show the expected modulation of ~2–4 kJ·mol^−1^ for CRPC-related aptamers [81,82]. Conventional SELEX cycles (12–15) are reduced to 5–7 in AI-assisted protocols [23,83,84,85,86,87], an approximate relative reduction of 42–58% (extremes: (12–5)/12 = 58.3%; (15–7)/15 = 53.3%). Purely computational studies sometimes report predicted gains without validated experimental K_d_ [88,89,90].

A focused subset of prostate-relevant affinity estimates, together with their methodological context, is summarized in (Table 7).

### 7.5. Diagnostic Performance (Se, Sp, AUC)

Complete 2 × 2 reporting (TP, FP, TN, FN) is uncommon; many studies report only AUC or Se/Sp without confidence intervals [19,21,22,23,24,27,28,29,39,68,69,70,71]. Urinary multiplex panels (PCA3, TMPRSS2:ERG, miR-21, miR-375, EN2) show point AUCs 0.70–0.92, with sensitivities up to 95% and specificities up to 88% (heterogeneous thresholds, often derivation-only). Insufficient comparable datasets (same biomarker, matrix, prespecified cut-off; threshold ≥ 5) precluded robust Reitsma bivariate meta-analysis; only limited clusters (e.g., PCA3, EN2) approach eligibility but differ in matrix (whole urine vs. uEV) and cut-off strategy (Youden vs. prespecified). Consequently, no HSROC summary is reported at this stage. When derived, likelihood ratios and DOR follow LR^+^ = Se/(1 − Sp); LR− = (1 − Se)/Sp; DOR = (LR+)/(LR−).

### 7.6. AI-SELEX: Operational and Translational Gains

AI integrations span sequence-structure classifiers, guided docking, generative models (reinforcement, diffusion, APIPred), and adaptive cycle stopping [23,83,84,85,87,95,96]. Outcomes: cycle reduction (cf. §7.4); affinity shifts toward lower K_d_ (typical Δlog_10_(K_d_) ≈ 0.3–1.0) in some comparative series (to be validated on final extraction; time compression via NGS integration, convergence-entropy metrics, and in silico parallelization) [85,87]. Gaps: (i) absent absolute K_d_, (ii) limited linkage between affinity improvements and diagnostic accuracy (few optimized sequences with AUC or paired Se/Sp).

### 7.7. Risk of Bias (QUADAS-2/PROBAST)

Patient selection: frequent high/unclear risk (single-center sampling, enriched case series, non-consecutive inclusion).

Index test: post hoc thresholds (Youden), blinding rarely reported.
Reference standard: typically histopathology; urine → biopsy intervals often unclear.
Flow and timing: undocumented post-inclusion exclusions; partial retesting.
Applicability: artificial matrices or spiked-urine validations elevate concern.

PROBAST (AI/multiparametric models): participants are often high risk (local series), non-standardized preprocessing of predictors, outcomes are sometimes composite or lack csPCa stratification, analysis is frequently missing calibration and external validation (optimism from internal CV only). ROBINS-I was not used to reclassify QUADAS-2 but aided contextual annotation.

### 7.8. Heterogeneity, Sensitivity Analyses, and Publication Bias

Major sources: matrix type (native urine vs. AUM vs. uEV) [8,9,75], assay maturity (prototype vs. clinical) [6,29], threshold strategy (post hoc vs. prespecified) [23,40], reference standard (biopsy vs. proxies) [68,71], and pre-analytics (post-DRE vs. spontaneous). Prespecified sensitivity analyses: exclusion of (i) studies with ≥2 QUADAS-2 high-risk domains, (ii) data restricted to artificial matrices without native-urine validation, (iii) approximate K_d_ (digitized; “*” in Table 6). Deeks’ test was not performed where there were <10 comparable datasets; no funnel plots generated.

### 7.9. Bibliometric Metadata Analyzed

The overall bibliometric indicators for the included corpus, summarized in (Table 8), indicate steady long-term growth with variable annual contributions. The distinct bibliometric corpus (n = 756) shows modest growth (CAGR 1.30%) from 28 (2010) to 34 (part of 2025), h-index 82, g-index 142, international co-authorship 25.26% [6,7,8,19,29,69,74,91,97,98,99,100,101]. High source dispersion (314) indicates an interdisciplinary field (biosensors, nanomaterials, molecular oncology). Recent output peaks in International Journal of Molecular Sciences and ACS Sensors reflect increasing sensor-biology integration.

### 7.10. Sensitivity Analyses, Heterogeneity, and Publication Bias Heterogeneity Sources

Matrix (native urine, artificial urine, buffer) [8,9,75], assay maturity (prototype vs. applied) [6,29], threshold strategy (Youden-derived vs. prespecified) [23,40], reference standards [68,71]. Sensitivity analyses were limited to clusters with ≥3 datasets, stratifying by urine source, validation type, exclusion of purely analytical systems [27,28,29,39]. Influence analyses required ≥4 datasets [69,70]. Deeks’ test was attempted only where ≥10 datasets existed [56,71]. Overall, aptamer-based urinary diagnostics show translational promise; AI-enhanced SELEX accelerates discovery, but heterogeneity, inconsistent reporting, and limited external validation remain barriers [6,7,8,9,10,11,19,21,22,23,24,27,28,29,39,68,69,70,71,72,73,74].

## 8. Discussion

### 8.1. Evidence Synthesis: AI-Driven SELEX as an Enabler of Urinary Multiplex Panels

This systematic review shows that integrating artificial intelligence into SELEX materially accelerates and refines the development of urinary aptamer panels for prostate cancer [102]. Conventional SELEX is hampered by lengthy enrichment cycles, stochastic clone fixation, and under-sampling of the chemically diverse urinary bi-omarker space [19,20]. AI approaches–including reinforcement learning, genetic algo-rithms, and sequence-structure predictors–enable rational triage of candidate pools, reducing experimental burden and achieving lower Kd values [22,23,103]. Beyond affinity optimization, AI facilitates multiplexing by retaining orthogonal aptamers across biomarker strata (lncRNAs, proteins, metabolites, exosomal miRNAs), reducing redundancy, and maximizing complementary diagnostic information [68,88,104,105]. For example, composite signatures integrating PCA3, TMPRSS2:ERG, EN2, and exosomal miRNAs were prioritized by AI-guided workflows [83,89,106]. Coupled bioinformatics in-terrogation of omics and extracellular vesicle repositories help ensure that target nomination reflects genuine tumor biology rather than analytical artifact [41,94,96,107]. Col-lectively, these findings support AI-SELEX as a translational enabler shifting urinary assays from isolated proof-of-concepts toward clinically oriented multiplex panels, with enrichment cycles consistently compressed from 12 to 15 to 5 to 7 (≈40–55% re-duction) [108,109,110,111,112].

Table 9 provides an integrative cross-walk linking methodological domains, analytical outputs, enabling technologies, and recurrent limitations across the included studies.

### 8.2. Limitations and Heterogeneity

Despite these advances, heterogeneity and methodological gaps temper the evi-dence. Pre-analytical protocols diverged substantially—some studies relied on native urine, others on synthetic/artificial matrices—without consistent control of collection, storage, or freeze–thaw cycles [18,23]. Many biosensor and affinity studies were based on small pilot cohorts (<50 cases/controls), limiting statistical power and external gen-eralizability [20,69,93]. Reporting gaps were frequent: selection cycles were incom-pletely described [23], absolute affinity values sometimes omitted [26], and validation often restricted to internal cross-validation [41]. Diagnostic performance reporting was also inconsistent: thresholds were variably Youden-optimized or ad hoc, calibra-tion rarely assessed [69], and complete 2 × 2 contingency data sparse. These inconsist-encies contributed to wide between-study variability in sensitivity, specificity, and AUC, constraining pooled analyses and complicating interpretation [113,114,115,116,117,118,119,120].

### 8.3. Clinical Implications and Implementation

From a clinical perspective, urinary aptamer diagnostics could reduce unnecessary biopsies, refine post-PSA risk stratification, and lower diagnostic costs [17]. Multiplex panels combining exosomal and soluble biomarkers promise enhanced specificity by mitigating false positives [3,22]. Implementation will require harmonized workflows: standardized urine handling, reproducible extracellular vesicle isolation, and biosensor outputs directly integrable with laboratory systems [3]. AI integration adds requirements for computational infrastructure, governance, and auditability [15,16,17], but it also compresses discovery cycles and reduces reagent-intensive wet-lab iterations [22]. Importantly, AI-SELEX outputs extend beyond diagnostics into theranostics: aptamers optimized for discrimination can be repurposed for targeted drug delivery or molecular imaging [121,122], reinforcing their dual clinical and economic value. As summarized in Table 9, cross-walk analysis highlights the main methodological domains, enabling features, and recurring limitations across the included studies

### 8.4. Methodological Recommendations

To accelerate translation, future research should prioritize methodological rigor. First, SELEX-AI workflows must adopt transparent reporting of library diversity, randomization, enrichment cycles, counter-selection steps, and computational filters [18,20,21]. Second, absolute affinity (K_d_) and kinetics should be reported consistently under defined assay conditions [23,57]. Third, diagnostic accuracy studies must provide full 2 × 2 contingency data, AUC with 95% confidence intervals, and calibration outputs to enable synthesis [123]. Fourth, external validation in multicenter, demographically diverse cohorts is essential to test generalizability [21]. Fifth, pre-registration of analytical protocols and analysis plans–including predefined thresholds–will reduce selective reporting [20]. Finally, multidisciplinary collaborations (computational scientists, molecular biologists, clinicians, engineers) are required to establish shared benchmarks such as minimal acceptable K_d_, cycle reduction targets, and validation standards [57]. Without these measures, high-potential prototypes risk stalling before clinical adoption.

### 8.5. Engineering-to-Clinical Performance Bridge

At equilibrium, the fractional occupancy of the aptamer–ligand complex is defined as:

$$\theta =\frac{\left[L\right]}{K_{d}+[L]}$$

where [L] is the free ligand concentration and $K_{d}$ the dissociation constant.

When $[L]\ll K_{d}$, $\theta \approx [L]/K_{d}$, indicating that reducing $K_{d}$ proportionally lowers the concentration needed for detectable binding, thus decreasing the analytical limit of detection (LoD) and improving sensitivity.

However, overall diagnostic accuracy (AUC, specificity) is not solely determined by affinity. It is strongly modulated by pre-analytical factors (e.g., urinary extracellular vesicles vs. whole urine; post-DRE vs. spontaneous sampling), non-specific binding events, and the use of pre-specified decision thresholds.

In practice, AI-assisted SELEX primarily enhances candidate prioritization and reduces the enrichment cycle burden (from 12 to 15 cycles to 5 to 7), thereby shaping the distribution of affinity and specificity across candidate pools, which indirectly influences clinical diagnostic performance.

## 9. Conclusions

This systematic and bibliometric review demonstrates that urinary aptamer as-says—particularly when integrated with extracellular vesicle (EV) fractions—offer a credible path to refine pre-biopsy stratification in prostate cancer. Across 129 included studies, single-marker assays such as PCA3 and EN2 achieved proof-of-concept diag-nostic performance, with limits of detection in the femtomolar to attomolar range [1,2,3,75,124,125]. Multiplex prototypes combining PCA3, EN2, PSA derivatives, sarcosine, and exosomal miRNAs provided complementary information streams but rarely pro-gressed beyond internal validation [7,9,36,65].

SELEX performance analysis highlighted AI-assisted workflows as a turning point. Compared with conventional 12–15 cycle protocols, machine-learning-driven priori-tization compressed enrichment to 5–7 cycles (≈40–55% relative reduction), delivered affinity gains up to 100-fold, and shortened calendar time through NGS-integrated stopping rules [23,25,66,96,126,127]. Yet, the translational bridge remains incomplete: many computationally optimized aptamers were not validated in clinical urine co-horts, and diagnostic endpoints such as AUC or calibration curves were underreported [19,21,27].

Taken together, evidence synthesis underscores a dual signal: (i) analytical feasi-bility—aptamers can reach clinically relevant detection thresholds in urine—and (ii) pipeline fragility—heterogeneity, missing 2 × 2 data, and lack of external validation currently limit regulatory translation.

Several priorities emerge for the next phase of urinary aptamer research. When it comes to urinary multi-cancer panels, the molecular diversity of urine supports ex-pansion beyond prostate cancer. Early studies have demonstrated aptamer binding to colorectal, ovarian, and bladder cancer vesicles [28,72,128], suggesting a multi-cancer urine panel is technically feasible. Comparative head-to-head benchmarks across tu-mor types will be critical to demonstrate added value over PSA-only pathways. Speaking of integration with MRI and AI workflows, radiogenomic pipelines already couple mpMRI with urinary signatures to stratify indolent versus aggressive disease. Embedding AI-SELEX multiplex panels into these decision algorithms could improve pre-biopsy specificity and reduce unnecessary sampling [10,11,12,13,14]. AI integration will also require infrastructure for reproducibility, version control, and regulatory audit. When it comes to open data and reproducibility, transparent deposition of SELEX se-quences, affinity constants, and diagnostic datasets into repositories such as OSF or Zenodo should become standard. Open-source benchmarks will allow reproducibility, reduce duplicative effort, and accelerate cross-cohort validation. Harmonized report-ing (PRISMA, QUADAS-2, PROBAST, SELEX-AI checklist) will support evidence syn-thesis and downstream guideline adoption, in line with recent community-level rec-ommendations on aptamer development [129].

AI-assisted SELEX is a promising strategy for accelerating high-affinity aptamer discovery and assembling multiplex urinary panels for prostate cancer, with consistent cycle reduction from 12 to 15 to 5 to 7 (≈40–55% relative reduction). Current evidence remains early phase, methodologically heterogeneous, and largely single center. Priori-ties include standardized uEV processing, complete 2 × 2 diagnostic reporting with confidence intervals, multicenter external validation, calibration and decision impact analyses, and harmonized LoD and Kd reporting frameworks. Without these, the promise of non-invasive, low-cost urinary aptamer diagnostics will remain underexploited; with them, the field is poised to deliver clinically actionable, globally scalable tools for precision oncology.

## Figures and Tables

**Table 1:** Bibliometric metadata of the analyzed corpus (n = 756).

Item	Value
Time span	2010–2025 (16 publication years; 2025 partial)
Data lock date	24 August 2025
Merged deduplicated records (Scopus + WoS)	892
Pre-analytical exclusions	136
Final analytical corpus	756
Distinct sources (journals/outlets)	314
Documents (2010/2025)	28/34
Compound annual growth rate (CAGR) 2010 to 2025	1.30%
Mean document age (ref. 2025)	6.58 years (median 6)
Total citations (as of data lock)	31,610
Mean citations per document	41.83 (median 21)
Total cited references	52,762
Mean references per document	69.78 (median 50)
Unique (disambiguated) authors	2956
Authorship occurrences	5066
Mean co-authors per document	6.70 (median 6)
Single-author documents	13
International collaboration (≥2 countries)	191 (25.26%)
Type—Research articles	488 (64.55%)
Type—Reviews	234 (30.95%)
Type—Book chapters	23 (3.04%)
Type—Conference papers	8 (1.06%)
Type—Short surveys	2 (0.26%)
Type—Book	1 (0.13%)
Corpus h-index	82
Corpus g-index	142

NOTE Table 1: The 756-document corpus (2010–part of 2025; data lock 24 August 2025) spans 314 distinct sources, with modest growth (CAGR 1.30%) from 28 documents in 2010 to 34 in 2025. At data lock, it accumulated 31,610 citations (mean 41.83; median 21) and 52,762 cited references (mean 69.78; median 50). A total of 2956 disambiguated authors generated 5066 authorship occurrences (mean 6.70 per document), with only 13 single-author papers. International collaborations constituted 25.26% of outputs. Research articles predominated (64.55%), followed by reviews (30.95%); other document types were marginal. The corpus h-index (82) and g-index (142) indicate a solidly cited core complemented by a concentrated subset of highly cited contributions.

**Table 2:** Full-text exclusion reasons by quality tier (Q1–Q3) and overall (n = 518).

Reason	Code	Q1 n (%)	Q2 n (%)	Q3 n (%)	Total n (%)
Secondary literature (reviews, overviews, non-primary)	DOC	196 (44.4%)	1 (33.3%)	33 (44.6%)	230 (44.4%)
Method/platform without clinical cohort	TECH	92 (20.9%)	2 (66.7%)	15 (20.3%)	109 (21.0%)
Out-of-scope population/matrix (non-prostate or non-urinary source)	POP	79 (17.9%)	0 (0.0%)	13 (17.6%)	92 (17.8%)
Therapeutic-only (no diagnostic metrics)	THER	29 (6.6%)	0 (0.0%)	5 (6.8%)	34 (6.6%)
Insufficient extractable diagnostic data (no sensitivity/specificity/AUC/LoD/K_d_)	DATA	23 (5.2%)	0 (0.0%)	4 (5.4%)	27 (5.2%)
Non-conforming index test (no aptamer or wrong matrix)	INDEX	19 (4.3%)	0 (0.0%)	3 (4.1%)	22 (4.2%)
Duplicate/overlapping cohort	DUP	3 (0.7%)	0 (0.0%)	1 (1.4%)	4 (0.8%)
Other (retraction, language barrier, inaccessible full text)	OTHER	0 (0.0%)	0 (0.0%)	0 (0.0%)	0 (0.0%)
Total		441 (100.0%)	3 (100.0%)	74 (100.0%)	518 (100.0%)

**Table 3:** Operational definitions of full-text exclusion categories.

Code	Category	Operational Definition/Rationale
DOC	Secondary literature	Non-primary articles (reviews, meta-analyses, perspectives, commentaries) without original cohort or experimental data.
TECH	Method/platform without clinical cohort	Technical developments/validations (e.g., sensors, omics platforms, SELEX optimization) without application to relevant clinical samples.
POP	Out-of-scope population/matrix	Studies outside prostate cancer, or using non-urinary matrices/non-urinary extracellular vesicles (uEVs) when urinary biomarkers were required.
THER	Therapeutic-only	Aptamers or related approaches evaluated only for delivery/modulation/treatment, without diagnostic metrics (sensitivity, specificity, AUC, LoD, K_d,_ or equivalents).
DATA	Insufficient extractable diagnostic data	Quantitative diagnostic data not extractable (no sensitivity, specificity, AUC, LoD, K_d,_ or equivalents).
INDEX	Non-conforming index test	Required aptamer absent or inadequate matrix (e.g., serum instead of urine).
DUP	Duplicate/overlapping cohort	Overlapping cohort with an included study, offering no added value (no new metrics).
OTHER	Other	Retracted articles, language barrier, or inaccessible full text despite reasonable attempts (e.g., unresolvable access issues).

**Table 4:** Annual publication trends and citation metrics of the included corpus.

Year	Documents	Total Citations	Mean Citations/Doc	Leading Authors (Co-Leaders)	Citations Leading Authors	Share Authors (%)	Leading Journal	Citations Journal	Share Journal (%)	Partial Year
2020	19	848	44.6	Hussain B.; Kulabhusan P. K.; Yüce M.	172	20.3	Int. J. Mol. Sci.	290	34.2	No
2021	24	483	20.1	Giangrande P. H.; Schrand B.; Shigdar S.; de Franciscis V.	83	17.2	Mol. Ther.	83	17.2	No
2022	30	294	9.8	Bilal M.; Er S.; Ebrahimi N.; Gelen S. S.; Hosseinikhah S. M.; Kyzas G. Z.; Mobashar A.; Rahdar A.; Sargazi S.	47	16.0	Chem.-Biol. Interact.	47	16.0	No
2023	26	181	7.0	Chen Z.; Lu J.; Ma Y.; Sun D.; Wu M.; Zhang L.	35	19.3	J. Pharm. Anal.	35	19.3	No
2024	24	179	7.5	Zhang Y.	60	33.5	Accounts Mater. Res.	33	18.4	No
2025	14	10	0.7	Hamdi F.; Hoseini S. J.; Roushani M.	6	60.0	Microchem. J.	6	60.0	Yes

**Table 5:** Analytical performance of urine-based aptamer biosensors (limits of detection, dynamic ranges).

Ref.	Biomarker	Platform	Assay Type	Matrix	LoD (M)	Range Min (M)	Range Max (M)	R^2^	Segment	Span (×)	Ratio Min/LoD
[75]	Sarcosine	EIS	COF-Aptamer MIP	Buffer	1.66 × 10^−13^	5.00 × 10^−13^	5.00 × 10^−11^	0.9972	1	1.00 × 10^2^	3.0
[75]	Sarcosine	EIS	COF-Aptamer MIP	Buffer	1.66 × 10^−13^	5.00 × 10^−11^	3.50 × 10^−10^	0.9974	2	7.0	3.01 × 10^2^
[76]	Sarcosine	Fluorescence	Nucleic amplification	Buffer	6.90 × 10^−9^	1.00 × 10^−8^	2.00 × 10^−6^	0.9951	1	2.00 × 10^2^	1.45
[76]	Sarcosine	Fluorescence	Nucleic amplification	AUM	6.90 × 10^−9^	1.00 × 10^−8^	2.00 × 10^−6^	0.9951	1	2.00 × 10^2^	1.45
[1]	EN2	SGFET	Graphene FET	Buffer	2.74 × 10^−18^	NR	NR	NR	1	NR	NR
[2]	PCA3	EIS	AuNP-Aptamer	Buffer	1.00 × 10^−15^	1.00 × 10^−11^	1.00 × 10^−9^	0.910	1	1.00 × 10^2^	1.00 × 10^4^
[2]	PCA3	EIS	AuNP-Aptamer	AUM	2.00 × 10^−14^	1.00 × 10^−13^	1.00 × 10^−9^	0.950	1	1.00 × 10^4^	5.0
[3]	PCA3	DPV	MB-Aptamer	Buffer	1.00 × 10^−13^	1.00 × 10^−13^	1.00 × 10^−8^	NR	1	1.00 × 10^5^	1.0
[3]	PCA3	DPV	MB-Aptamer	AUM	1.00 × 10^−13^	1.00 × 10^−13^	1.00 × 10^−8^	NR	1	1.00 × 10^5^	1.0
[4]	Sarcosine	Electrochemical	Oxide nanosheet	Urine (unspecified)	3.50 × 10^−13^	1.00 × 10^−12^	8.00 × 10^−6^	NR	1	8.00 × 10^6^	2.86
[5]	EN2	ELONA	HCR	Buffer	3.40 × 10^−10^	3.90 × 10^−10^	2.50 × 10^−8^	0.991	1	64.1	1.15
[5]	EN2	ELONA	HCR	AUM	2.69 × 10^−9^	3.12 × 10^−9^	5.00 × 10^−8^	0.971	1	16.0	1.16

NOTE Table 5: LoD method reported as IUPAC 3σ/slope unless specified; NR = not reported.

**Table 6:** Binding affinity of selected aptamers (K_d_, log_10_(K_d_), ΔG at 298 K, and assay T).

Ref.	Target	Context	Platform	Assay	K_d_ (nM)	K_d_ (M)	log10 K_d_ (M)	ΔG_298K (kJ·mol^−1^)	ΔG_Tassay (kJ·mol^−1^)	T_assay (K)	Confidence	Notes
[82]	CRda8	CRPC cells	Flow cytometry	Cell binding	4.9	4.90 × 10^−9^	−8.310	−47.4	−44.1	277	High	4 °C
[82]	CRda8	CRPC cells	Flow cytometry	Cell binding	101.3	1.013 × 10^−7^	−6.994	−39.9	−41.5	310	High	37 °C
[82]	CRda21	CRPC cells	Flow cytometry	Cell binding	33.3	3.33 × 10^−8^	−7.478	−42.7	−39.7	277	High	4 °C
[82]	CRda21	CRPC cells	Flow cytometry	Cell binding	62.7	6.27 × 10^−8^	−7.203	−41.1	−42.8	310	High	37 °C
[29]	Androgen receptor element	Nucleic element	ELASA	Plate	5.5	5.50 × 10^−9^	−8.260	−47.1	NR	NR	High	-
[91]	PSA variant 1	Protein	SPR	Binding	177.0	1.77 × 10^−7^	−6.752	−38.5	NR	NR	High	-
[91]	PSA variant 2	Protein	SPR	Binding	357.0	3.57 × 10^−7^	−6.447	−36.8	NR	NR	High	-
[92]	Spheroid aptamer A4	3D spheroid	Flow cytometry	3D binding	72.0 *	7.20 × 10^−8^	−7.143	−40.7	NR	NR	Approx	Digitized
[93]	Wy5a	PC3 cells	Flow cytometry	Cell binding	73.6	7.36 × 10^−8^	−7.133	−40.7	NR	NR	High	-
[93]	Wy5b	PC3 cells	Flow cytometry	Cell binding	173.1	1.731 × 10^−7^	−6.762	−38.6	NR	NR	High	-
[94]	AMC51 (AMACR)	Protein	ELASA	Plate	49.0	4.90 × 10^−8^	−7.310	−41.7	NR	NR	High	Range 0.5–500 nM
[94]	AMC55 (AMACR)	Protein	ELASA	Plate	140.0	1.40 × 10^−7^	−6.854	−39.1	NR	NR	High	-
[94]	AMC56 (AMACR)	Protein	ELASA	Plate	66.0	6.60 × 10^−8^	−7.180	−41.0	NR	NR	High	Interval 66–73
[3]	PCA3 sensor	Target transcript	Electrochemistry	DPV	30.0 *	3.00 × 10^−8^	−7.523	−42.9	NR	NR	Approx	Digitized

Notes Table 6: Thermodynamic relation: $\Delta G=R\cdot T\cdot ln(K_{d}).$ Constants: $R=8.314\times 10^{-3}kJ{mol}^{-1}K^{-1}$; standard state 1 M (ln = natural logarithm). Assay temperature: 277 K (4 °C) or 310 K (37 °C) if explicitly reported; otherwise NR (ΔG_Tassay). Rows marked “*” (Approx) = values digitized from figures; excluded from high-confidence sensitivity analyses. No ionic activity correction; ΔG conditional on experimental buffers. Rounding differences ≤ 0.1 $kJ{mol}^{-1}$ vs. prior versions (systematic harmonization).

**Table 7:** Comparative subset of aptamer binding-affinity data with selected targets and assay conditions.

Ref.	Target	Context	Platform	Assay	K_d_ (nM)	K_d_ (M)	log10 K_d_ (M)	ΔG_298K (kJ·mol^−1^)	ΔG_Tassay (kJ·mol^−1^)	T_assay (K)	Confidence	Notes
[82]	CRda8	CRPC cells	Flow cytometry	Cell binding	4.9	4.90 × 10^−9^	−8.310	−47.4	−44.1	277	High	4 °C
[82]	CRda8	CRPC cells	Flow cytometry	Cell binding	101.3	1.013 × 10^−7^	−6.994	−39.9	−41.5	310	High	37 °C
[29]	Androgen receptor element	Nucleic element	ELASA	Plate	5.5	5.50 × 10^−9^	−8.260	−47.1	NR	NR	High	-
[91]	PSA variant 1	Protein	SPR	Binding	177.0	1.77 × 10^−7^	−6.752	−38.5	NR	NR	High	-
[93]	Wy5a	PC3 cells	Flow cytometry	Cell binding	73.6	7.36 × 10^−8^	−7.133	−40.7	NR	NR	High	-
[94]	AMC51 (AMACR)	Protein	ELASA	Plate	49.0	4.90 × 10^−8^	−7.310	−41.7	NR	NR	High	Range 0.5–500 nM

**Table 8:** Summary of core bibliometric indicators for the analyzed corpus (n = 756).

Item	Value
Time span	2010–2025 (partially)
Final analytical corpus	756
Distinct sources	314
Research articles	64.6%
Reviews	31.0%
h-index	82
g-index	142
International collaborations	25.3%
CAGR (2010–2025)	1.30%

**Table 9:** Cross-walk of methodological domains, measured outcomes, enabling features, and recurring gaps.

Method/Domain	What Was Measured in Our Corpus	AI/Engineering Features Enabling Gains	Recurring Limitations
Electrochemical aptasensors	Limit of detection down to 2.74 × 10^−18^ M for EN2 (buffer); PCA3 10^−15^ → 2 × 10^−14^ M with broad ranges; calibration often R^2^ ≥ 0.90.	Graphene transistors, gold nanoparticles, microfluidic handling; introduction of RangeMin/LoD ratio to assess effective sensitivity.	Measurements often in buffer or artificial urine instead of native urine; small pilot cohorts; incomplete diagnostic data with confidence intervals; thresholds frequently derived post hoc; few external validations.
Optical and enzymatic amplification assays	For EN2, limit of detection 3.40 × 10^−10^ M (buffer) and 2.69 × 10^−9^ M (artificial urine); calibration R^2^ ≈ 0.97.	Hybridization chain reaction and nucleic-acid cascades enabling portable optical detection.	Matrix effects insufficiently characterized; robustness in native urine rarely assessed; turnaround time and cost not reported.
Exosome and extracellular vesicle capture assays	Recovery and fraction-specific detection, including microRNAs (miR-21, miR-375) and proteins such as EN2.	Aptamer-mediated capture (CD63, CD9), microfluidic immunocapture, hybrid capture plus signal amplification.	Heterogeneous pre-analytical conditions (digital rectal exam vs. spontaneous sampling; isolation protocols); inter-laboratory reproducibility underreported.
SELEX with affinity readouts	Dissociation constants typically from high picomolar to hundreds of nanomolar; log_10_(K_d_ [M]) and Gibbs free energy at 298 K calculated; thermal shifts of ~2–4 kJ·mol^−1^ confirmed at 277 K and 310 K.	Counter-selection against benign matrices; chemical modifications (fluoro, LNA); cycle compression from 12 to 15 to 5 to 7.	Dissociation constants sometimes missing or approximate; kinetic parameters (association/dissociation rates) seldom reported; many results limited to buffer-only conditions.
AI-guided SELEX and sequence design	When validated, affinity gains of Δlog_10_(K_d_) ≈ 0.3–1.0; cycle reduction from 12 to 15 to 5 to 7.	Machine learning models, reinforcement learning stopping rules, genetic algorithms, generative approaches, sequencing feedback loops.	Many predicted sequences not tested in clinical urine; weak linkage between affinity improvements and diagnostic accuracy; limited transparency on training and testing sets.
Multiplex biomarker panels	Single studies report area under the curve between 0.70 and 0.92, sensitivities up to 95%, specificities up to 88%.	Integration of multiple fractions (cells, soluble, vesicles); alignment with imaging pathways.	Very few datasets for pooled analysis; results often derivation-only; calibration and decision curve analyses lacking; external validation minimal.
Validation and reporting standards	Area under the curve with 95% confidence intervals; likelihood ratios and diagnostic odds ratio when full data were available.	Emphasis on pre-registration and prespecified cut-offs.	Calibration uncommon; thresholds often not predefined; variation in biopsy standards and timing; publication bias tests rarely feasible.
Implementation and cost	Bench-to-result times variably reported; cost per test seldom explicit.	Development of cartridge-ready sensors; automation of vesicle isolation; integration with laboratory systems.	Few studies explicitly reported turnaround time or cost; future work must address these parameters.

## Data Availability

Data supporting the findings of this study are available within the article and its Supplementary Materials. The bibliometric dataset (n = 756 references) and extraction template (n = 129 studies) are available on request from the corresponding author.

## Supplementary Materials

The following supporting information can be downloaded at https://www.mdpi.com/article/10.3390/biomedicines13122877/s1, Table S1: PRISMA 2020 flow diagram of study selection. Detailed reasons for full-text exclusions (n = 518): VOSviewer bibliometric mapping workflow (keyword co-occurrence and clustering).

## Abbreviations

The following abbreviations are used in this manuscript:

TLA	Three-Letter Acronym
EV	Extracellular Vesicle
SELEX	Systematic Evolution of Ligands by Exponential Enrichment
AI	Artificial Intelligence
PCa	Prostate Cancer
LoD	Limit of Detection
AUC	Area Under the Curve
HSROC	Hierarchical Summary Receiver Operating Characteristic

## References

[1] Dong J., Yang H., Song P., Yu T., Pan Z., Chen Z., Wang R., Deng M., Wang X., Li J. (2025). Highly Sensitive Detection of Engrailed-2 Protein Biomarker in Urine for Using Solution-Gated Graphene Transistor Diagnosis of Prostate Cancer. ACS Sens..

[2] Takita S., Nabok A., Mussa M., Kitchen M., Lishchuk A., Smith D. (2024). Ultrasensitive prostate cancer marker PCA3 detection with impedimetric biosensor based on specific label-free aptamers. Biosens. Bioelectron. X.

[3] Takita S., Nabok A., Lishchuk A., Mussa M.H., Smith D. (2023). Enhanced Performance Electrochemical Biosensor for Detection of Prostate Cancer Biomarker PCA3 Using Specific Aptamer. Eng.

[4] Farokhi S., Roushani M., Saedi Z. (2023). Fabrication of an electrochemical aptasensor for the determination of sarcosine based on synthesized CuCo_2_O_4_ nanosheets. Anal. Methods.

[5] Kim E., Kang M., Ban C. (2022). Aptamer-antibody hybrid ELONA that uses hybridization chain reaction to detect a urinary biomarker EN2 for bladder and prostate cancer. Sci. Rep..

[6] Takita S., Nabok A., Lishchuk A., Mussa M.H., Smith D. (2022). Detection of Prostate Cancer Biomarker PCA3 with Electrochemical Apta-Sensor ^†^. Eng. Proc..

[7] Cheng H., Yang Q., Wang R., Luo R., Zhu S., Li M., Li W., Chen C., Zou Y., Huang Z. (2022). Emerging Advances of Detection Strategies for Tumor-Derived Exosomes. Int. J. Mol. Sci..

[8] Sun D., Ma Y., Wu M., Chen Z., Zhang L., Lu J. (2023). Recent progress in aptamer-based microfluidics for the detection of circulating tumor cells and extracellular vesicles. J. Pharm. Anal..

[9] Farshchi F., Hasanzadeh M. (2020). Nanomaterial based aptasensing of prostate specific antigen (PSA): Recent progress and challenges in efficient diagnosis of prostate cancer using biomedicine. Biomed. Pharmacother..

[10] Banerjee J., Nilsen-Hamilton M. (2013). Aptamers: Multifunctional molecules for biomedical research. J. Mol. Med..

[11] Chang Y.M., Donovan M.J., Tan W. (2013). Using aptamers for cancer biomarker discovery. J. Nucleic Acids.

[12] Sefah K., Bae K.-M., Phillips J.A., Siemann D.W., Su Z., McClellan S., Vieweg J., Tan W. (2013). Cell-based selection provides novel molecular probes for cancer stem cells. Int. J. Cancer.

[13] Hu M., Zhang K. (2013). The application of aptamers in cancer research: An up-to-date review. Future Oncol..

[14] Liu K., Lin B., Lan X. (2013). Aptamers: A promising tool for cancer imaging, diagnosis, and therapy. J. Cell Biochem..

[15] Yi K., Rong Y., Huang L., Tang X., Zhang Q., Wang W., Wu J., Wang F. (2021). Aptamer-Exosomes for Tumor Theranostics. ACS Sens..

[16] Janas T., Janas P., Sapoń K., Janas T. (2020). Binding of rna aptamers to membrane lipid rafts: Implications for exosomal mirnas transfer from cancer to immune cells. Int. J. Mol. Sci..

[17] Xie S., Sun W., Fu T., Liu X., Chen P., Qiu L., Qu F., Tan W. (2023). Aptamer-Based Targeted Delivery of Functional Nucleic Acids. J. Am. Chem. Soc..

[18] Feng X., Zhu Y., Wang F., Guo T., Dou X., Lin M., Tian W. (2022). The Aptamer Functionalized Nanocomposite Used for Prostate Cancer Diagnosis and Therapy. J. Nanomater..

[19] Zhu L., Zhao J., Guo Z., Liu Y., Chen H., Chen Z., He N. (2021). Applications of aptamer-bound nanomaterials in cancer therapy. Biosensors.

[20] Li Z., Fu X., Huang J., Zeng P., Huang Y., Chen X., Liang C. (2021). Advances in Screening and Development of Therapeutic Aptamers Against Cancer Cells. Front. Cell Dev. Biol..

[21] Fu Z., Xiang J. (2020). Aptamer-functionalized nanoparticles in targeted delivery and cancer therapy. Int. J. Mol. Sci..

[22] Jeevanandam J., Tan K.X., Danquah M.K., Guo H., Turgeson A. (2019). Advancing Aptamers as Molecular Probes for Cancer Theranostic Applications—The Role of Molecular Dynamics Simulation. Biotechnol. J..

[23] Pang X., Cui C., Wan S., Jiang Y., Zhang L., Xia L., Li L., Li X., Tan W. (2018). Bioapplications of cell-SELEX-generated aptamers in cancer diagnostics, therapeutics, theranostics and biomarker discovery: A comprehensive review. Cancers.

[24] Nimjee S.M., White R.R., Becker R.C., Sullenger B.A. (2017). Aptamers as Therapeutics. Annu. Rev. Pharmacol. Toxicol..

[25] Zhang L., Wan S., Jiang Y., Wang Y., Fu T., Liu Q., Cao Z., Qiu L., Tan W. (2017). Molecular Elucidation of Disease Biomarkers at the Interface of Chemistry and Biology. J. Am. Chem. Soc..

[26] Chandola C., Kalme S., Casteleijn M.G., Urtti A., Neerathilingam M. (2016). Application of aptamers in diagnostics, drug-delivery and imaging. J. Biosci..

[27] Ozalp V.C., Kavruk M., Dilek O., Bayrac A.T. (2015). Aptamers: Molecular tools for medical diagnosis. Curr. Top. Med. Chem..

[28] Huang J., Chen X., Fu X., Li Z., Huang Y., Liang C. (2021). Advances in Aptamer-Based Biomarker Discovery. Front. Cell Dev. Biol..

[29] Thevendran R., Tang T.-H., Citartan M. (2023). In-silico selection employing rigid docking and molecular dynamic simulation in selecting DNA aptamers against androgen receptor. Biotechnol. J..

[30] Kelly L., Maier K.E., Yan A., Levy M. (2021). A comparative analysis of cell surface targeting aptamers. Nat. Commun..

[31] An J., Park H., Kim J., Park H., Kim T.-H., Park C., Kim J., Lee M.-H., Lee T. (2023). Extended-Gate Field-Effect Transistor Consisted of a CD9 Aptamer and MXene for Exosome Detection in Human Serum. ACS Sens..

[32] Cha B.S., Jang Y.J., Lee E.S., Kim D.Y., Woo J.S., Son J., Kim S., Shin J., Han J., Kim S. (2023). Development of a Novel DNA Aptamer Targeting Colorectal Cancer Cell-Derived Small Extracellular Vesicles as a Potential Diagnostic and Therapeutic Agent. Adv. Healthc. Mater..

[33] Hanžek A., Ducongé F., Siatka C., Duc A.-C.E. (2023). Identification and Characterization of Aptamers Targeting Ovarian Cancer Biomarker Human Epididymis Protein 4 for the Application in Urine. Cancers.

[34] Liu Y., Hu B., Pei X., Li J., Qi D., Xu Y., Ou H., Wu Y., Xue L., Huang J.H. (2023). A Non-G-Quadruplex DNA Aptamer Targeting NCL for Diagnosis and Therapy in Bladder Cancer. Adv. Healthc. Mater..

[35] Sun X., Xie L., Qiu S., Li H., Zhou Y., Zhang H., Zhang Y., Zhang L., Xie T., Chen Y. (2022). Elucidation of CKAP4-remodeled cell mechanics in driving metastasis of bladder cancer through aptamer-based target discovery. Proc. Natl. Acad. Sci. USA.

[36] Song Z., Mao J., Barrero R.A., Wang P., Zhang F., Wang T. (2020). Development of a cd63 aptamer for efficient cancer immunochemistry and immunoaffinity-based exosome isolation. Molecules.

[37] Wang Y., Zhang Y., Li P.-C., Guo J., Huo F., Yang J., Jia R., Wang J., Huang Q., Theodorescu D. (2022). Development of Novel Aptamer-Based Targeted Chemotherapy for Bladder Cancer. Cancer Res..

[38] Roy D., Pascher A., Juratli M.A., Sporn J.C. (2021). The potential of aptamer-mediated liquid biopsy for early detection of cancer. Int. J. Mol. Sci..

[39] Díaz-Fernández A., Lorenzo-Gómez R., Miranda-Castro R., de-los-Santos-Álvarez N., Lobo-Castañón M.J. (2020). Electrochemical aptasensors for cancer diagnosis in biological fluids—A review. Anal. Chim. Acta.

[40] Ram T.B., Krishnan S., Jeevanandam J., Danquah M.K., Thomas S. (2024). Emerging Biohybrids of Aptamer-Based Nano-Biosensing Technologies for Effective Early Cancer Detection. Mol. Diagn. Ther..

[41] Mamidi N., De Silva F.F., Vacas A.B., Gutiérrez Gómez J.A., Montes Goo N.Y., Mendoza D.R., Reis R.L., Kundu S.C. (2024). Multifaceted Hydrogel Scaffolds: Bridging the Gap between Biomedical Needs and Environmental Sustainability. Adv. Health. Mater..

[42] Zhong J., Ding J., Deng L., Xiang Y., Liu D., Zhang Y., Chen X., Yang Q. (2021). Selection of DNA aptamers recognizing epcam-positive prostate cancer by cell-SELEX for in vitro and in vivo MR imaging. Drug Design. Dev. Ther..

[43] Campos-Fernández E., Barcelos L.S., Souza A.G., Goulart L.R., Alonso-Goulart V. (2020). Post-SELEX Optimization and Characterization of a Prostate Cancer Cell-Specific Aptamer for Diagnosis. ACS Omega.

[44] Huang Z.-X., Xie Q., Guo Q.-P., Wang K.-M., Meng X.-X., Yuan B.-Y., Wan J., Chen Y.-Y. (2017). DNA aptamer selected for specific recognition of prostate cancer cells and clinical tissues. Chin. Chem. Lett..

[45] Duan M., Long Y., Yang C., Wu X., Sun Y., Li J., Hu X., Lin W., Han D., Zhao Y. (2016). Selection and characterization of DNA aptamer for metastatic prostate cancer recognition and tissue imaging. Oncotarget.

[46] Grover R., Drall S., Poonia N., Kumar Jain G., Aggarwal G., Lather V., Kesharwani P., Pandita D., Goyal R.K. (2022). CD44 and CD133 aptamer directed nanocarriers for cancer stem cells targeting. Eur. Polym. J..

[47] Maghsoudi S., Shahraki B.T., Rabiee N., Afshari R., Fatahi Y., Dinarvand R., Ahmadi S., Bagherzadeh M., Rabiee M., Tahriri M. (2019). Recent Advancements in aptamer-bioconjugates: Sharpening Stones for breast and prostate cancers targeting. J. Drug Deliv. Sci. Technol..

[48] Huang C.-P., Hu W.-P., Yang W., Lee Z.-J., Chen W.-Y. (2025). In silico maturation of DNA aptamer against the prostate-specific antigen (PSA) and kinetic analysis. Biochem. Biophys. Res. Commun..

[49] Bağda E., Bağda E., Liu J. (2025). A Fluorescent Aptasensor for Sensitive and Selective Determination of Epigenetic Cancer Biomarker N1-Methyladenosine in Urine Samples. Chem.-A Eur. J..

[50] Sun P., Gou H., Che X., Chen G., Feng C. (2024). Recent advances in DNAzymes for bioimaging, biosensing and cancer therapy. Chem. Commun..

[51] Trivedi J., Yasir M., Maurya R.K., Tripathi A.S. (2024). Aptamer-based Theranostics in Oncology: Design Strategies and Limitations. BIO Integr..

[52] Yu X., Yang H., Huang X. (2018). Novel Method for Structure-Activity Relationship of Aptamer Sequences for Human Prostate Cancer. ACS Omega.

[53] Fasogbon I.V., Ondari E.N., Tusubira D., Rangasamy L., Venkatesan J., Musyoka A.M., Aja P.M. (2024). Recent focus in non-SELEX-computational approach for de novo aptamer design: A mini review. Anal. Biochem..

[54] Yu X., Yu R., Tang L., Guo Q., Zhang Y., Zhou Y., Yang Q., He X., Yang X., Wang K. (2014). Recognition of candidate aptamer sequences for human hepatocellular carcinoma in SELEX screening using structure-activity relationships. Chemom. Intell. Lab. Syst..

[55] Kar R.K. (2024). High-throughput and computational techniques for aptamer design. Expert Opin. Drug Discov..

[56] Kabir A., Bhattarai M., Peterson S., Najman-Licht Y., Rasmussen K.Ø., Shehu A., Bishop A.R., Alexandrov B., Usheva A. (2024). DNA breathing integration with deep learning foundational model advances genome-wide binding prediction of human transcription factors. Nucleic Acids Res..

[57] Wang Z., Liu Z., Zhang W., Li Y., Feng Y., Lv S., Diao H., Luo Z., Yan P., He M. (2024). AptaDiff: De novo design and optimization of aptamers based on diffusion models. Briefings Bioinform..

[58] Li Y., Tam W.W., Yu Y., Zhuo Z., Xue Z., Tsang C., Qiao X., Wang X., Wang W., Li Y. (2023). The application of Aptamer in biomarker discovery. Biomark. Res..

[59] Sousa D.A., Carneiro M., Ferreira D., Moreira F.T.C., Sales M.G.F., Rodrigues L.R. (2022). Recent Advances in the Selection of Cancer-Specific Aptamers for the Development of Biosensors. Curr. Med. Chem..

[60] Razlansari M., Jafarinejad S., Rahdar A., Shirvaliloo M., Arshad R., Fathi-Karkan S., Mirinejad S., Sargazi S., Sheervalilou R., Ajalli N. (2023). Development and classification of RNA aptamers for therapeutic purposes: An updated review with emphasis on cancer. Mol. Cell Biochem..

[61] Rana S., Kaushik D., Singh A., Gautam D., Rai J., Rathore J.S. (2023). Aptamer: A theranostic approach towards breast cancer. Clin. Immunol. Commun..

[62] Janani S.K., Dhanabal S.P., Sureshkumar R., Upadhyayula S.S.N. (2022). Anti-nucleolin Aptamer as a Boom in Rehabilitation of Breast Cancer. Curr. Pharm. Des..

[63] Esposito C.L., Quintavalle C., Ingenito F., Rotoli D., Roscigno G., Nuzzo S., Thomas R., Catuogno S., de Franciscis V., Condorelli G. (2021). Identification of a novel RNA aptamer that selectively targets breast cancer exosomes. Mol. Ther. Nucleic Acids.

[64] Liu Y., Wang Z., Zhuo Y., Wu H., Peng Y., Wang T., Peng T., Qiu L., Tan W. (2024). Aptamer-Based Multiparameter Analysis for Molecular Profiling of Hematological Malignancies. Anal. Chem..

[65] Adachi T., Nakamura S., Michishita A., Kawahara D., Yamamoto M., Hamada M., Nakamura Y. (2024). RaptGen-Assisted Generation of an RNA/DNA Hybrid Aptamer against SARS-CoV-2 Spike Protein. Biochemistry.

[66] Shelley G., Dai J., Keller J.M., Keller E.T. (2021). Pheno-SELEX: Engineering anti-metastatic aptamers through targeting the invasive phenotype using systemic evolution of ligands by exponential enrichment. Bioengineering.

[67] Tran T.T.T., Delgado A., Jeong S. (2021). Organ-on-a-Chip: The Future of Therapeutic Aptamer Research?. Biochip J..

[68] Zhou G., Wilson G., Hebbard L., Duan W., Liddle C., George J., Qiao L. (2016). Aptamers: A promising chemical antibody for cancer therapy. Oncotarget.

[69] Sun H., Zhu X., Lu P.Y., Rosato R.R., Tan W., Zu Y. (2014). Oligonucleotide aptamers: New tools for targeted cancer therapy. Mol. Ther. Nucleic Acids.

[70] Tran P.H.-L., Xiang D., Nguyen T.N.-G., Tran T.T.-D., Chen Q., Yin W., Zhang Y., Kong L., Duan A., Chen K. (2020). Aptamer-guided extracellular vesicle theranostics in oncology. Theranostics.

[71] Liu Y., Zhu P., Huang J., He H., Ma C., Wang K. (2022). Integrating DNA nanostructures with DNAzymes for biosensing, bioimaging and cancer therapy. Coord. Chem. Rev..

[72] Campos-Fernández E., Oliveira Alqualo N., Moura Garcia L.C., Coutinho Horácio Alves C., Ferreira Arantes Vieira T.D., Caixeta Moreira D., Alonso-Goulart V. (2021). The use of aptamers in prostate cancer: A systematic review of theranostic applications. Clin. Biochem..

[73] Shi X., Chen L., Chen S., Sun D. (2021). Electrochemical aptasensors for the detection of hepatocellular carcinoma-related biomarkers. New J. Chem..

[74] Barbas A.S., Mi J., Clary B.M., White R.R. (2010). Aptamer applications for targeted cancer therapy. Future Oncol..

[75] Hamdi F., Roushani M., Hoseini S.J. (2024). Novel biosensor for sarcosine detection in prostate cancer: Combining molecular imprinted polymer and aptamer strategies. Microchem. J..

[76] Yuan L., Liu X., Yan H., Jiang B., Yuan R., Xiang Y. (2024). Cascaded and autocatalytic nucleic acid circuit for exponentially amplified and aptamer-based ultrasensitive fluorescent biosensing of sarcosine cancer biomarker. Sens. Actuators B Chem..

[77] Esposito C.L., Quintavalle C., Ingenito F., Rotoli D., Roscigno G., Nuzzo S., Thomas R., Catuogno S., Minic Z., Berezovski M. (2021). Targeting breast cancer exosomes with nucleic aptamers: Innovative tools for early diagnosis and therapy. Sib. Med Rev..

[78] Long Z., Bing T., Zhang N., Zu S., Sheng J., Zhang X., Liu X., Shangguan D. (2025). DNA aptamer targeting zinc transporters ZIP10 and ZIP6 on cancer cells. Talanta.

[79] Kim M.W., Kwon S.-H., Choi J.H., Lee A. (2018). A promising biocompatible platform: Lipid-based and bio-inspired smart drug delivery systems for cancer therapy. Int. J. Mol. Sci..

[80] Turetta M., Del Ben F., Brisotto G., Biscontin E., Bulfoni M., Cesselli D., Colombatti A., Scoles G., Gigli G., Del Mercato L.L. (2018). Emerging technologies for cancer research: Towards personalized medicine with microfluidic platforms and 3D tumor models. Curr. Med. Chem..

[81] Domenyuk V., Zhong Z., Stark A., Xiao N., O’Neill H.A., Wei X., Wang J., Tinder T.T., Tonapi S., Duncan J. (2017). Plasma Exosome Profiling of Cancer Patients by a Next Generation Systems Biology Approach. Sci. Rep..

[82] Zhong J., Liu D., Yang Q., Ding J., Chen X. (2024). A Novel DNA Aptamer Probe Recognizing Castration Resistant Prostate Cancer in vitro and in vivo Based on Cell-SELEX. Drug Des. Dev. Ther..

[83] Kong H.Y., Byun J. (2015). Screening and Characterization of a Novel RNA Aptamer That Specifically Binds to Human Prostatic Acid Phosphatase and Human Prostate Cancer Cells. Mol. Cells.

[84] Cha B.S., Lee E.S., Kim J., Son J., Kim D., Kim S., Park K.S. (2025). Aptamer-directed tyramide signal amplification for ultrasensitive detection of small extracellular vesicles for temporally heterogenous colorectal cancer. Sens. Actuators B Chem..

[85] Mohammadinejad A., Gaman L.E., Aleyaghoob G., Gaceu L., Mohajeri S.A., Moga M.A., Badea M. (2024). Aptamer-Based Targeting of Cancer: A Powerful Tool for Diagnostic and Therapeutic Aims. Biosensors.

[86] Manea I., Casian M., Hosu-Stancioiu O., de-los-Santos-Álvarez N., Lobo-Castañón M.J., Cristea C. (2024). A review on magnetic beads-based SELEX technologies: Applications from small to large target molecules. Anal. Chim. Acta.

[87] Song S., Wang X., Xu K., Li Q., Ning L., Yang X. (2019). Selection of highly specific aptamers to Vibrio parahaemolyticus using cell-SELEX powered by functionalized graphene oxide and rolling circle amplification. Anal. Chim. Acta.

[88] Platella C., Riccardi C., Montesarchio D., Roviello G.N., Musumeci D. (2017). G-quadruplex-based aptamers against protein targets in therapy and diagnostics. Biochim. Biophys. Acta-Gen. Subj..

[89] Mathew A., Maekawa T., Sakthikumar D. (2015). Aptamers in targeted nanotherapy. Curr. Top. Med. Chem..

[90] Sekhon S.S., Ahn G., Park G.-Y., Park D.-Y., Lee S.-H., Ahn J.-Y., Kim Y.-H. (2019). The Role of Aptamer Loaded Exosome Complexes in the Neurodegenerative Diseases. Toxicol. Environ. Health Sci..

[91] Díaz-Fernández A., Miranda-Castro R., de-los-Santos-Álvarez N., Rodríguez E.F., Lobo-Castañón M.J. (2019). Focusing aptamer selection on the glycan structure of prostate-specific antigen: Toward more specific detection of prostate cancer. Biosens. Bioelectron..

[92] Souza A.G., Marangoni K., Fujimura P.T., Alves P.T., Silva M.J., Bastos V.A.F., Goulart L.R., Goulart V.A. (2016). 3D Cell-SELEX: Development of RNA aptamers as molecular probes for PC-3 tumor cell line. Exp. Cell Res..

[93] Wang Y., Luo Y., Bing T., Chen Z., Lu M., Zhang N., Shangguan D., Gao X. (2014). DNA aptamer evolved by cell-SELEX for recognition of prostate cancer. PLoS ONE.

[94] Yang D.-K., Chen L.-C., Lee M.-Y., Hsu C.-H., Chen C.-S. (2014). Selection of aptamers for fluorescent detection of alpha-methylacyl-CoA racemase by single-bead SELEX. Biosens. Bioelectron..

[95] Fang Y., Lin S., Yang F., Situ J., Lin S., Luo Y. (2020). Aptamer-Conjugated Multifunctional Polymeric Nanoparticles as Cancer-Targeted, MRI-Ultrasensitive Drug Delivery Systems for Treatment of Castration-Resistant Prostate Cancer. BioMed Res. Int..

[96] Drabik A., Ner-Kluza J., Mielczarek P., Civit L., Mayer G., Silberring J. (2018). Advances in the Study of Aptamer-Protein Target Identification Using the Chromatographic Approach. J. Proteome Res..

[97] Cunha P.D.S., de Miranda M.C., de Melo M.I.A., Ferreira A.D.F., Barbosa J.L., Oliveira J.A.D.C., Goes T.D.S., Gomes D.A., de Goes A.M. (2024). Selection of internalizing RNA aptamers into human breast cancer cells derived from primary sites. J. Cell Biochem..

[98] Pereira A.C., Pina A.F., Sousa D., Ferreira D., Santos-Pereira C., Rodrigues J.L., Melo L.D.R., Sales G., Sousa S.F., Rodrigues L.R. (2022). Identification of novel aptamers targeting cathepsin B-overexpressing prostate cancer cells. Mol. Syst. Des. Eng..

[99] Pandey P.N., Saini N., Sapre N., Kulkarni D.A., Tiwari D.A.K. (2021). Prioritising breast cancer theranostics: A current medical longing in oncology. Cancer Treat. Res. Commun..

[100] Bing T., Wang J., Shen L., Liu X., Shangguan D. (2020). Prion Protein Targeted by a Prostate Cancer Cell Binding Aptamer, a Potential Tumor Marker?. ACS Appl. Bio Mater..

[101] Orava E.W., Cicmil N., Gariépy J. (2010). Delivering cargoes into cancer cells using DNA aptamers targeting internalized surface portals. Biochim. Biophys. Acta-Biomembr..

[102] Speransky S., Serafini P., Caroli J., Bicciato S., Lippman M.E., Bishopric N.H. (2019). A novel RNA aptamer identifies plasma membrane ATP synthase beta subunit as an early marker and therapeutic target in aggressive cancer. Breast Cancer Res. Treat..

[103] Zhang L., Martini G.D., Tomas Rube H., Kribelbauer J.F., Rastogi C., FitzPatrick V.D., Houtman J.C., Bussemaker H.J., Pufall M.A. (2018). SelexGLM differentiates androgen and glucocorticoid receptor DNA-binding preference over an extended binding site. Genome Res..

[104] Mercier M.-C., Dontenwill M., Choulier L. (2017). Selection of nucleic acid aptamers targeting tumor cell-surface protein biomarkers. Cancers.

[105] Chen M., Yu Y., Jiang F., Zhou J., Li Y., Liang C., Dang L., Lu A., Zhang G. (2016). Development of cell-SELEX technology and its application in cancer diagnosis and therapy. Int. J. Mol. Sci..

[106] Mattice C.M.C., DeRos M.C. (2015). Status and Prospects of Aptamers as Drug Components. BioDrugs.

[107] Hornung T., O’Neill H.A., Logie S.C., Fowler K.M., Duncan J.E., Rosenow M., Bondre A.S., Tinder T., Maher V., Zarkovic J. (2021). ADAPT identifies an ESCRT complex composition that discriminates VCaP from LNCaP prostate cancer cell exosomes. Nucleic Acids Res..

[108] Nguyen P.-L., Sekhon S.S., Ahn J.-Y., Ko J.H., Lee L., Cho S.-J., Min J., Kim Y.-H. (2017). Aptasensor for environmental monitoring. Toxicol. Environ. Health Sci..

[109] Berg K., Lange T., Mittelberger F., Schumacher U., Hahn U. (2016). Selection and Characterization of an α6β4 Integrin blocking DNA Aptamer. Mol. Ther. Nucleic Acids.

[110] Ye M., Hu J., Peng M., Liu J., Liu J., Liu H., Zhao X., Tan W. (2012). Generating aptamers by cell-SELEX for applications in molecular medicine. Int. J. Mol. Sci..

[111] Wu J., Wang C., Li X., Song Y., Wang W., Li C., Hu J., Zhu Z., Li J., Zhang W. (2012). Identification, Characterization and Application of a G-Quadruplex Structured DNA Aptamer against Cancer Biomarker Protein Anterior Gradient Homolog 2. PLoS ONE.

[112] Ni X., Castanares M., Mukherjee A., Lupold S.E. (2011). Nucleic acid aptamers: Clinical applications and promising new horizons. Curr. Med. Chem..

[113] Tan W., Wang H., Chen Y., Zhang X., Zhu H., Yang C., Yang R., Liu C. (2011). Molecular aptamers for drug delivery. Trends Biotechnol..

[114] Kanwar J.R., Roy K., Kanwar R.K. (2011). Chimeric aptamers in cancer cell-targeted drug delivery. Crit. Rev. Biochem. Mol. Biol..

[115] Meyer C., Hahn U., Rentmeister A. (2011). Cell-specific aptamers as emerging therapeutics. J. Nucleic Acids.

[116] Wang J., Li G. (2011). Aptamers against cell surface receptors: Selection, modification and application. Curr. Med. Chem..

[117] Rialon K.L., White R.R. (2011). Aptamers: Potential applications to pancreatic cancer therapy. Anti-Cancer Agents Med. Chem..

[118] Li L., Wang W., Xu X., Wang H., Liao S., Li W., Zhang W., Liu D., Cao B., Wang S. (2011). Aptamer-based radioimmunotherapy: The feasibility and prospect in cancer therapy. J. Radioanal. Nucl. Chem..

[119] Lee J.H., Yigit M.V., Mazumdar D., Lu Y. (2010). Molecular diagnostic and drug delivery agents based on aptamer-nanomaterial conjugates. Adv. Drug Deliv. Rev..

[120] Ray P., White R.R. (2010). Aptamers for targeted drug delivery. Pharmaceuticals.

[121] Wen X., Huang Z., Yang X., He X., Li L., Chen H., Wang K., Guo Q., Liu J. (2024). Development of an aptamer capable of multidrug resistance reversal for tumor combination chemotherapy. Proc. Natl. Acad. Sci. USA.

[122] Yoon S., Rossi J.J. (2018). Therapeutic potential of small activating RNAs (saRNAs) in human cancers. Curr. Pharm. Biotechnol..

[123] Karlsen K.K., Wengel J. (2012). Locked nucleic acid and aptamers. Nucleic Acid Ther..

[124] Jeong S., Han S.R., Lee Y.J., Lee S.-W. (2010). Selection of RNA aptamers specific to active prostate-specific antigen. Biotechnol. Lett..

[125] Gong S., Ren H., Lin C., Hu P., Tian R., Liu Z., Li Y., Zhou Y., Yang Y., Lu S. (2018). Immunochromatographic strip biosensor for the rapid detection of N-glycolylneuraminic acid based on aptamer-conjugated nanoparticle. Anal. Biochem..

[126] Ferreira L., Flanagan S.P., Fogel R., Limson J.L. (2024). Generation of epitope-specific hCG aptamers through a novel targeted selection approach. PLoS ONE.

[127] Li W., Yan H., Wang H., Zhao M., Zhang S. (2025). In Vitro Selection of Aptamers Against CD64 and Unraveling of the Molecular Mechanisms of Aptamer-Target Interactions. Indian J. Microbiol..

[128] Vandghanooni S., Sanaat Z., Barar J., Adibkia K., Eskandani M., Omidi Y. (2021). Recent advances in aptamer-based nanosystems and microfluidics devices for the detection of ovarian cancer biomarkers. TrAC Trends Anal. Chem..

[129] Toulmé J.-J., Giangrande P.H., Mayer G., Suess B., Ducongé F., Sullenger B., De Franciscis V., Darfeuille F., Peyrin E. (2017). Aptamers in Bordeaux, 24–25 June 2016. Pharmaceuticals.

